# Chronic Microdose Lithium Treatment Prevented Memory Loss and Neurohistopathological Changes in a Transgenic Mouse Model of Alzheimer's Disease

**DOI:** 10.1371/journal.pone.0142267

**Published:** 2015-11-25

**Authors:** Marielza Andrade Nunes, Natalia Mendes Schöwe, Karla Cristina Monteiro-Silva, Ticiana Baraldi-Tornisielo, Suzzanna Ingryd Gonçalves Souza, Janaina Balthazar, Marilia Silva Albuquerque, Ariadiny Lima Caetano, Tania Araujo Viel, Hudson Sousa Buck

**Affiliations:** 1 Department of Physiological Sciences, Santa Casa de São Paulo School of Medical Sciences, R. Dr. Cesario Motta Junior, 61, 11° andar, São Paulo, SP 01221–020, Brazil; 2 Graduation Course on Pharmacology, Institute of Biomedical Sciences, Universidade de São Paulo, Avenida Professor Lineu Prestes, 1524, 05508–900 São Paulo, Brazil; 3 School of Arts, Sciences and Humanities, Universidade de São Paulo, Av. Arlindo Bettio, 1000, São Paulo, SP 03828–080, Brazil; 4 Research Group on Neuropharmacology of Aging—ReGNA, Sao Paulo, Brazil; University of Lancaster, UNITED KINGDOM

## Abstract

The use of lithium is well established in bipolar disorders and the benefits are being demonstrated in neurodegenerative disorders. Recently, our group showed that treatment with microdose lithium stabilized the cognitive deficits observed in Alzheimer’s disease (AD) patients. In order to verify the lithium microdose potential in preventing the disease development, the aim of this work was to verify the effects of chronic treatment with microdose lithium given before and after the appearance of symptoms in a mouse model of a disease similar to AD. Transgenic mice (Cg-Tg(PDGFB-APPSwInd)20Lms/2J) and their non-transgenic litter mate genetic controls were treated with lithium carbonate (1.2 mg/Kg/day in drinking water) for 16 or 8 months starting at two and ten months of age, respectively. Similar groups were treated with water. At the end of treatments, both lithium treated transgenic groups and non-transgenic mice showed no memory disruption, different from what was observed in the water treated transgenic group. Transgenic mice treated with lithium since two months of age showed decreased number of senile plaques, no neuronal loss in cortex and hippocampus and increased BDNF density in cortex, when compared to non-treated transgenic mice. It is suitable to conclude that these data support the use of microdose lithium in the prevention and treatment of Alzheimer’s disease, once the neurohistopathological characteristics of the disease were modified and the memory of transgenic animals was maintained.

## Introduction

Demographic changes resulting from the increase in life span and the increasing number of aged people lead to a dramatic increase in the prevalency of dementias. Among them, Alzheimer’s disease is a progressive neurodegenerative disease, whose ethiology is still unknown, and causes progressive cognitive deficit and incapacity. Aging is the most important risk factor, suggesting that biological alterations related to aging may be associated to its pathogenesis [1], besides genetic and environmental factors. Normally, patients present progressive atrophy of gray matter, which can be a reflection of neuronal death caused by micro-structural changes in brain [2].

Gradual accumulation of neurofibrillary tangles, mainly intracellularly, formed as a result of abnormal hyperphosphorylation of cytoskeletal tau protein and deposition of amyloid-β peptide fibrils into senile plaques, mainly extracellularly, are the most important histopathological characteristics of AD that lead to huge neuronal death [3]. The biochemical mechanisms that are activated to produce these alterations are many and are evidently present in specific vulnerable brain areas. One of the brain areas most succeptible is the hippocampus [4].

Recently, our research team showed that treatment with microdose lithium carbonate (1.5 mg/day) was efficient to prevent the cognitive decline of patients with clinical diagnosis for AD [5]. Although these exciting data in humans, there is no evidence of the efficacy of this microdose as a preventive strategy. In the same way, the ability to modify the disease properties was not measured yet. The neuroprotective mechanisms of lithium have already been described [6,7] and the benefits of lithium involve inhibition of GSK-3β leading to decreased tau phosphorylation and to the decrease of amyloid-β (Aβ) load [8,9]. Lithium may also protect neurons against the neurotoxic effects of Aβ42 by favoring other neurotrophic and/or neuroprotective responses not only by GSK3 inhibition. Another important neuroprotective effect of lithium is the stimulation of synthesis and release of neurotrophins, in particular brain-derived neurotrophic factor (BDNF) and vascular endothelial growth factor (VEGF) [7]. Therefore, lithium treatment may provide an array of benefits that could lead to a global improvement in the organism function.

However, it is already known that lithium could be toxic in weight-based dosing [10], mainly in aged people. So, the aim of this work was to investigate the preventive and therapeutic effects of microdose lithium in a mouse model of neurodegenerative disease and to explore their molecular mechanisms. This work is the first to show that continuous preventive, as well as continuous therapeutic treatment with microdose lithium can alter the pathological characteristics of Alzheimer’s disease, preventing its evolution. In this way, this work gives support for the clinical use of microdose lithium to prevent and stabilize the progression of the disease.

## Materials and Methods

### 1. Animals

Male hemizygous transgenic mice—here called TG—B6.Cg-Tg (PDGFB-APPSwInd)—that express a mutant form of the human amyloid precursor protein (hAPP) carrying the Swedish (K670N, M671L) and Indiana (V717F) familial AD mutations directed by the platelet-derived growth factor (PDGF) β-chain promoter (APP mice, J20 line) and age-matched wild type litter mates—here called WT—were provided from our own colony, using breeding males acquired from “The Jackson Laboratories”, USA (Stock Number 006293) [11]. To ensure the presence of the mutant gene, all animals were genotyped following the protocol described by the Jackson Laboratory. Between 5 and 7 months of age, these animals show diffuse deposition of amyloid-β (Aβ) peptide in dentate gyrus and neocortex and show senile plaques at 8 to 10 months of age displaying the full spectrum of cerebrovascular, cognitive, neuroinflammatory and amyloid pathologies at 10 months of age [11,12,13,14]. Animals from the same litter were maintained in number of 3 to 6, in individually ventilated cages, with food and water *ad libitum*. Room temperature was 24°C to 26°C and humidity was 55%.

All efforts were done to reduce the number of animals and their suffering. The experimental procedings were performed according to the “ethics principles for the use of laboratory animals” described by the Brazilian Society in Science of Laboratory Animals (SBCAL, Brazil). The experimental protocols were approved by the Animal Ethics Committee from Santa Casa de São Paulo School of Medical Sciences, under number 013/11.

### 2. Treatment

Animals were divided in groups of transgenic and non-transgenic animals treated or not with lithium. Two groups of animals were treated from 2 months of age until 18 months of age (TG Li16 and WT Li16). Other two groups of animals were treated from 10 months of age until 18 months of age (TG Li8 and WT Li8). Two other groups did not receive any treatment (TG Ctrl and WT Ctrl). Lithium (in the form of Lithium Carbonate) was dissolved in water and administered *ad libitum*. The dose used was proportional to that used in a previous manuscript from our group [5], i.e., 1.5 mg of lithium carbonate/day, which corresponds to 0.006 mEq of lithium/Kg. This dose was adjusted according to mice pharmacokinetics [15,16], with the dose correction index for humans multiplied by 10 [16]. The final dose was 0.005 mg/day/animal, which corresponds to 0.25 mg/kg.

### 3. Behavioural Tests

#### 3.1. Evaluation of Locomotor Activity

Spontaneous locomotor activity was measured as described earlier [17,18], using an animal activity cage (model 7430, Ugo Basile, Comerio, Italy). The apparatus consisted of a transparent acrylic cage (35 cm x 23 cm x 20 cm) with a set of horizontal sensors to register locomotor activity and a set of vertical sensors to register standing activity. At 6 and 18 months of age, each animal was placed alone inside the cage, and locomotion was immediately recorded for 5 min. During this period, immobility and grooming were also recorded.

#### 3.2. Evaluation of Spatial Memory

Spatial memory was evaluated using the Barnes maze, according to previous studies [19]. Briefly, the equipment was constituted by a white arena (diameter 100 cm) positioned 1 m from the floor. The arena had 30 holes (5 cm diameter each), disposed radially and a black cardboard wall around its circumference. The wall had four yellow figures to add spatial orientation and, above the center of the arena, a fluorescent lamp was placed. The illumination created an uncomfortable environment that motivated the animals to escape from the arena to a dark box placed under one of the holes, filled with bedding material. In the training session, each mouse was placed in the center of the arena under a round acrylic box for 1 min. After that, the animal was released and the time to find the escape box was registered. The maximal time allowed for the exploration was 5 min. If the animal did not find the correct hole, it was gently conducted to the hole by the experimenter. Once inside the box, the animal was left there for 5 min. Just after, all the protocols were repeated once more. In the next 5 days (test days), twice a day, each mouse was placed inside the round acrylic box for 1 min and left to explore the arena until it found the escape box or for a maximum of 5 min. The escape box was placed always in the same hole for the same animal but in different holes among animals. The maze was cleaned using a 5% ethanol solution before the test of each animal.

Latency to find the escape box and the distance covered were assessed. Spatial memory was evaluated every 3 months, as described in “Experimental Design”. In graphs, only the results related to the 1^st^ day of each evaluation were plotted.

#### 3.3. Evaluation of anxiety

The anxiety behaviour was evaluated when the animals were 18 months old, using an elevated plus maze. The equipment was composed of two open arms (50 x 10 cm) and two closed arms with the same dimensions, with 40 cm hight walls. The maze was maintained 50 cm over the floor. Animals were left in experimental room for one hour before the beginning of observations. After this period, each one was placed in the center of the maze and the behavior was registered for 5 minutes. Time spent in open and closed arms as well as number of entries in each arm were recorded and presented as the ratio of parameters between open and closed arms.

Animals’ performance was recorded with a JVC Everio videocamera and analyzed with Smart Software, version 2.5.21 (Panlab Harvard Apparatus).

#### 3.4. Evaluation of aversive-related memory

To evaluate this type of memory an inhibitory avoidance shuttle box was used (Ugo Basile, Italy). The box has two chambers, a dark and a light one, separated by a guillotine-door. The method is based on the animal’s aversion for light places and its preference for close and dark places. On the acquisition session, the animal was placed in the light chamber. After 2 seconds the door was opened and the animal was left to explore the box. When the animal entered the dark chamber, the door was closed and the animal received an electrical stimulus, on paws, of 0.5 mA for 2 s. Time spent to get in the dark chamber was recorded. After that, animal was returned to its home cage. Maximal time for animals staying in the box was 5 min.

On the test session, 24 h later, the animal was placed again in the dark side of the box and the time spent to enter in the dark chamber was recorded again. The greater the latency, the better the animal’s memory. Aversive-related memory was assessed when animals were 18 months old.

### 4. Time Line of Experimental Design

**Fig 1:**
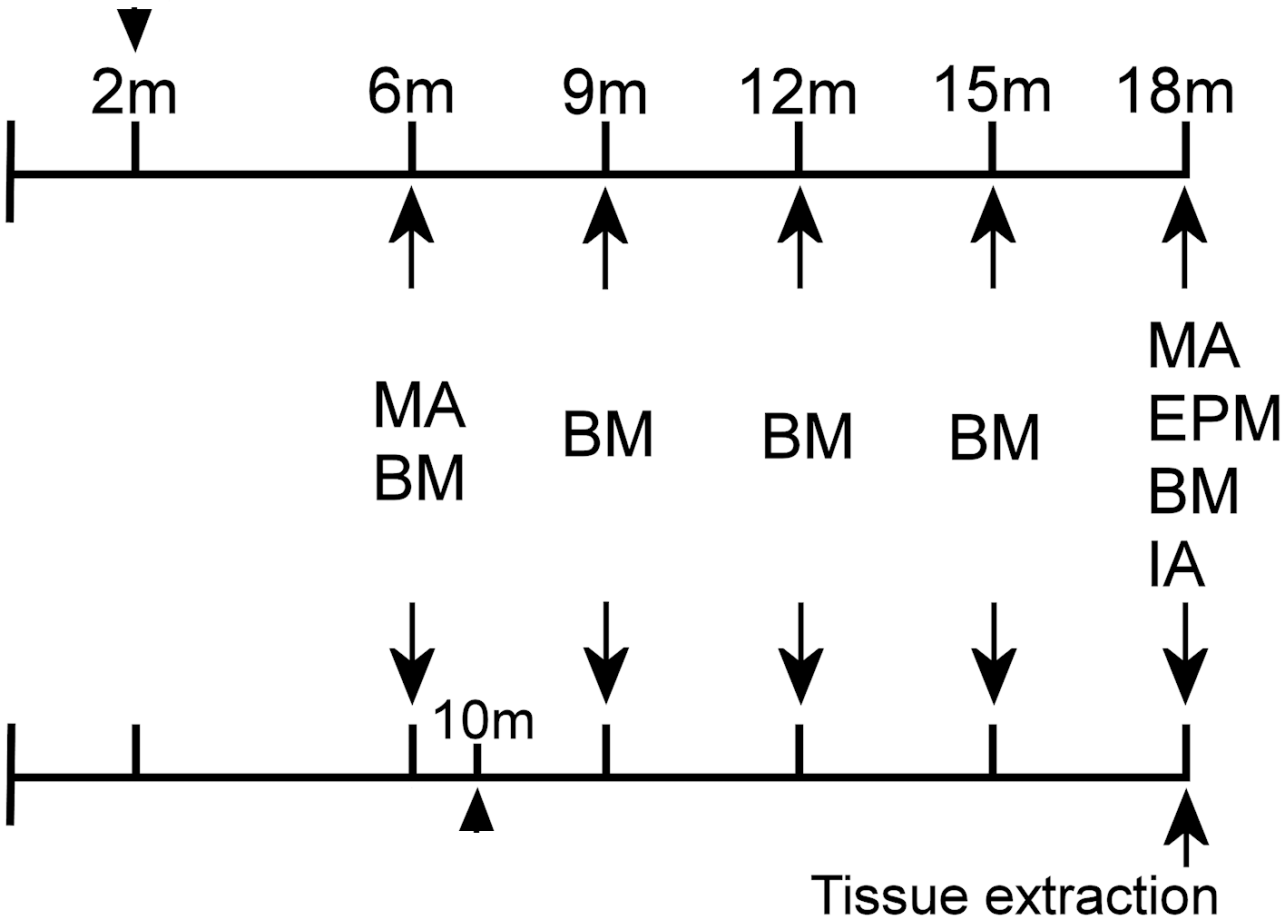
Time-line of the experimental protocol. Animals were treated from 2 or 10 months of age (arrow heads). Behavioral experiments (arrows) began when all animals were 6 months old and were repeated every 3 months. When all animals were 18 months old, the last behavioral observations were done, animals were killed and brain tissue was extracted. MA: motor activity; BM: Barnes maze; EPM: elevated plus maze; IA: inhibitory avoidance.

Treatment of WT16 and TG16 began when animals were 2 months old whereas treatment of WT8 and TG8 began when mice were 10 months old (Fig 1). At 6 months of age, animals were submitted to evaluation of locomotion and spatial memory. During all the treatment time, spatial memory was evaluated every 3 months. At 18 months of age, besides the evaluation of locomotor activity and spatial memory, anxiety and aversive-related memory were also verified. After the behavioural observations, animals were killed and the brains were collected for histological and molecular analysis (Fig 1).

### 5. Histological and biochemical analysis

After the behavioural tests, animals were anesthetized with isofluorane and brains were removed, immediately frozen in 2-dimethylbutane (-45°C to -55°C) and stored at -80°C until used. Half of each brain was used to histological analysis, so samples were cut (20 μm) in a cryostat (−20°C to −22°C, Microm HM525, Germany) and sections were mounted on gelatin-coated slides, were desiccated for 5 min at room temperature and were kept at −80°C until use. The other hemisphere was used to western-blot and ELISA analysis.

#### 5.1. Evaluation of neuronal bodies density

The number of cellular bodies was evaluated in the dentate gyrus (DG), CA1, CA2 and CA3 of the hippocampus and the frontal cortex, using an antibody for the protein NeuN (ABN78, Millipore, USA). Frozen slides containing brain samples were warmed to room temperature for ± 10 min and then fixed with 100% acetone at -20°C, at room temperature, for 5 min. The sections were washed in PBS and endogenous peroxidase activity was blocked with 0.6% hydrogen peroxide in methanol for 20 min, and the sections were washed again in PBS for 5 min. Non-specific sites were blocked by incubating with goat serum (normal serum) for 30 min and incubated with primary antibody against NeuN (1:2000) for 2 h at room temperature. Then slides were washed in PBS for 5 min and incubated with secondary antibody conjugated with biotin produced in the same serum as used for the blocking step for 1 h at room temperature. After incubation, the sections were processed with the avidin/biotin blocking kit (Vectastain Elite ABC Kit—Vector Laboratories Inc., USA) for 30 min and washed with PBS for 5 min. The immune complexes were detected using diaminobenzidine (DAB) solution. Finally, slides were washed twice with water and were dehydrated through alcohol gradient starting at 75% ethanol up to 95% and 100% ethanol for 5 min each. Then the slides were soaked twice in xylene for 5 min and mounted under coverslips. A microcomputer-based image analysis system (MCID; Imaging Research, Interfocus Europe, UK) coupled to a Nikon Eclipse E-600 microscope was used to acquire and calculate the proportion of the stained area to the total test area (proportional area = stained area/total tested area). Data were analized by the area occupied by positive DAB reactions. For the background control, slices incubated only with the secondary antibody were used. Each animal had their own slide for background control. Images were acquired in RGB and the range of detection of positive immunolabelings was set for each animal. In this way, all the positive DAB reactions were considered. Light levels were unchanged in slides of a batch. Values from each section were averaged for each animal, and a mean was determined across animals. The person that made the measurements was blind to the treatment.

#### 5.2. Quantification of senile plaques

Brain samples (obtained as described above) were warmed to room temperature (22°C) and were air dried (5–10 min). Slides were washed 5 times in PBS and incubated in Thioflavine S solution 0.1% in PBS, containing 0.1%Triton, for 5 min. After that, sections were washed twice with PBS, incubated with ethanol 70% for 5 min and washed, again, 3 times with PBS. At the end, sections were covered with coverslips using Fluoroshield with DAPI (Sigma, USA). All the process was done in a dark room. Analysis of senile plaques labeled with Thioflavine S was performed using an optic microscope (Nikon Eclipse E-600), with suitable filter for fluorescence. Pictures of plaques were taken just after the labeling, counted manually and analysed dividing the number of plaques by the number of sections analysed.

#### 5.3. Evaluation of synaptophysin density and GSK-3β activity

Cortical and hippocampal tissues were homogenized in lysis buffer containing 50 mM Tris–HCl (pH 7.4), 0.1% Triton X-100, 4 mM ethylene glycol tetraacetic acid (EGTA), 10 mM ethylenediamine tetracetic acid (EDTA), a tablet with protease inhibitors (Complete EDTA-free, Roche Diagnostics, Germany) and a phosphatase inhibitor cocktail tablet (PhosStop, Roche Diagnostics, Germany). The supernatant fractions were subsequently centrifuged at 12,000 g for 15 min, at 4°C, to remove debris, and total protein contain was determined according to previous published method [20]. Proteins (7 or 50 μg/lane, according to the protein of interest) were separated by 10% sodium dodecyl sulfate-polyacrylamide gel electrophoresis (SDS-PAGE) and transferred onto nitrocellulose membranes. To ensure equal protein loading, the Ponceau method was used to stain the membranes before probing with the antibodies [21]. For Synaptophysin and GSK-3β, membranes were preincubated with Tris (100 mM, pH 7.6) containing 0.1% Tween-20 and 0.9% saline (TBST) and 5% non fat milk for 1 h. For phosphorylated GSK-3β (pGSK-3β), membranes were blocked overnight with 5% bovine serum albumin (BSA) in TBST. After washing, membranes were incubated overnight at 4°C with primary polyclonal rabbit synaptophysin (abcam ab68851, 1:3000), GSK-3β (abcam ab18893, 1:200) or phosphoGSK-3β (abcam ab131097, 1:1000) antibodies.

The membranes were washed and then incubated with a secondary biotinylated-antibody horseradish peroxidase-conjugated Goat-anti-Rabbit IgG, diluted 1:4000 in TBST for 2 hours at room temperature. The immune complexes were detected by chemiluminescence, using a digital image system (ImageQuant^™^ LAS 500, GE, Sweden). After deteccion, membranes were stripped with stripping buffer (200 mM glycine, 0.1% SDS, 1% Tween-20, pH 2.2) and then incubated with β-tubulin antibody (abcam ab134185, 1:6000). The density of immunoblotting was quantified with image J software [22]. Data were reported in relation to the intensity of β-tubulin band. The activity of GSK-3β enzyme was determined as a ratio of pGSK-3β/GSK-3β.

#### 5.4. Determination of BDNF density

To determine BDNF content in cortex and hippocampus, it was used the ELISA Sandwich kit (BDNF Emax^®^ ImmunoAssay System, Promega, USA). Tissues samples were homogenized in lysis buffer 20 mM Tris-HCl, pH 8,0 containing 137 mM NaCl, 1% Triton-X, 10% glycerol and a tablet with protease inhibitors (Complete EDTA-free, Roche Diagnostics) and the assay was processed according to manufacturer’s instruction.

### 6. Statistical analysis

Behavioural data obtained in Barnes maze were expressed as means ± standard errors and analysed by repeated measures two-way ANOVA followed by Bonferroni post-hoc test.

Data obtained in motor activity cage were expressed as means ± standard errors and analysed by two-way ANOVA followed by Bonferroni post-hoc test.

Data obtained in elevated plus maze were expressed as means ± standard errors and analysed by one-way ANOVA followed by Tukey’s multiple comparison test.

Data obtained in inhibitory avoidance shuttle box were expressed by medians and interquartile ranges and analysed with two-way ANOVA followed by Holm-Sidak’s multiple comparison test. When appropriate, comparison between WT Ctrl and TG Ctrl groups were made using Student-t test.

Data related to all other experiments were analysed by Student-t test or one-way ANOVA followed by Tukey’s multiple comparison test, when aplicable. Since brain samples were divided in order to allow histological and molecular analysis, in some experiments the number of replicates (n) were on the low side. In this way, data were analyzed using Kruskal-Wallis non-parametric one-way ANOVA followed by Dunn’s multiple comparison test.

All analysis were done using StatSoft, Inc. (2013) STATISTICA (data analysis software system), version 12, www.statsoft.com. Values were considered significant when P < 0.05.

## Results and Discussion

### 1. General observations

The motivation of this work to investigate the preventive and therapeutic effects of microdose lithium in transgenic mice for AD, focusing on its molecular effects, was based on previously reported neuroprotective mechanisms of lithium and its toxicity in weight-based dosing [6,10], mainly in the elderly. Moreover, this work follows on previous results with the microdose of lithium (1.5 mg/day) as a stabilizer of cognitive loss in patients diagnosed with Alzheimer’s disease (AD) [5].

Lithium was administered as lithium carbonate in the dose 1.5 mg/day. The concentration of lithium to be dissolved in water during all the observation time, as long as the necessity of dose adjustment during the period, were determined according to mice water consumption. Detection of a significant diference in the ingested volume of water of treated versus untreated animals is important to advise possible renal toxicity, which will lead to increases in water comsumption [23]. However, no significant difference was observed in water ingestion between lithium treated and untreated groups (data not shown).

Toxicity was also monitored by mortality index (number of deaths/total number of animals) and animals’ age at death. No difference among groups were detected (P = 0.2349, data not shown). Among WT mice, only two animals died between 12 and 18 months of age, indenpendent on the treatment they were submitted to. In TG group, there were 3 deaths among TG Ctrl, one death among TG Li16 animals (between 15 and 18 months of age) and no deaths among TG Li8 group.

### 2. Mobility

**Fig 2:**
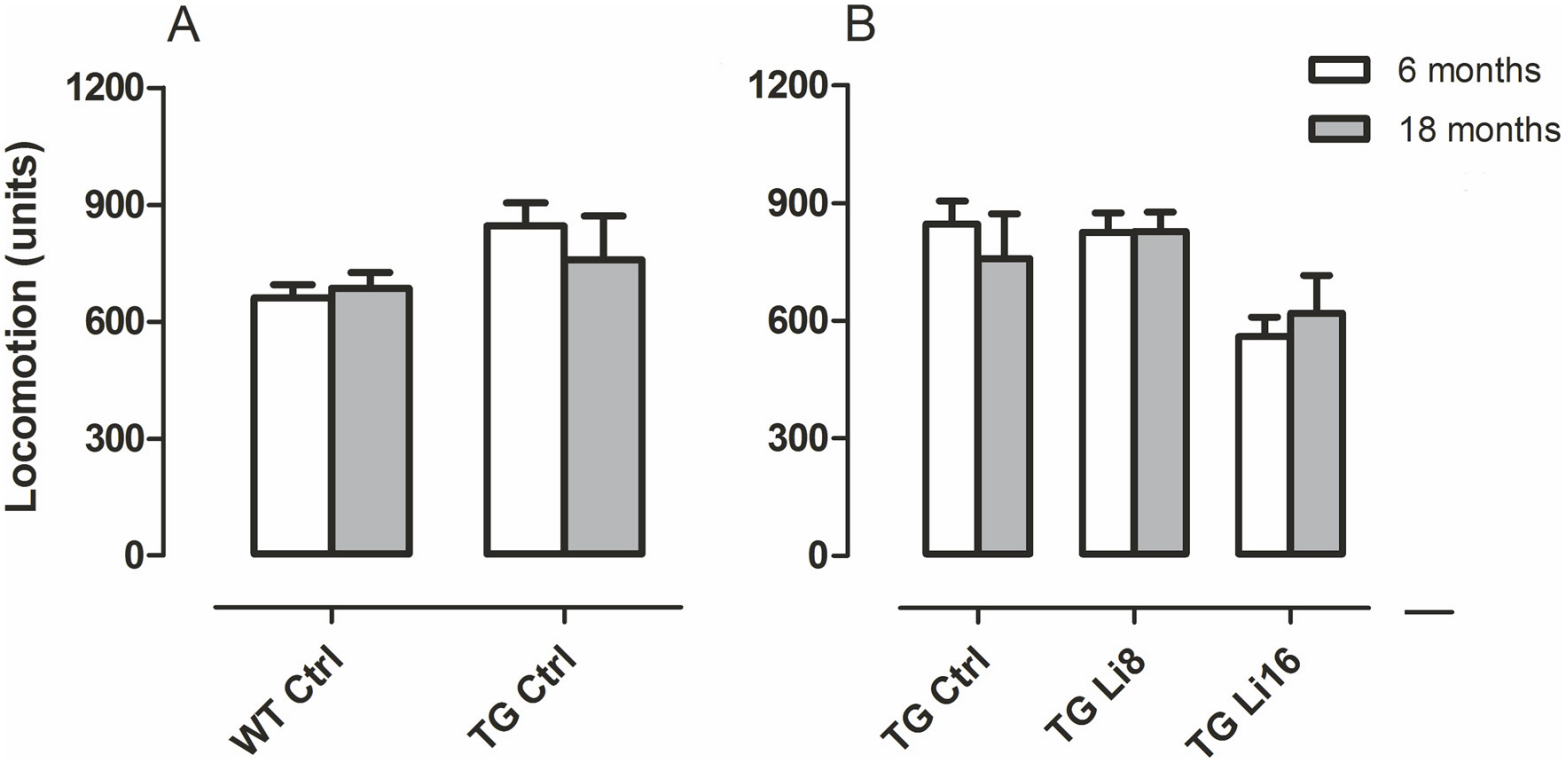
Treatment with lithium did not interfere in motor activity of the animals, both WT and TG. There was no difference in locomotion when WT and TG mice were compared, at 6 (WT, n = 10; TG, n = 7) and 18 months-old (WT, n = 10; TG, n = 7) (A). In the first evaluation, at 6 months, TG group presented greater rearing behavior than WT (B). Between the transgenic groups, we observed no difference in locomotion in both ages analyzed (C), but TG Ctrl (n = 7) showed more rearing behavior than TG Li8 (n = 9) and TG Li16 (n = 7), at 6 months of age. Data are plotted as mean ± SEM of arbitrary units. * P<0.05.

The behavioural tests used in this project depend on good locomotor activity, as animals need to explore the mazes and equipment to have their behavior evaluated. In order to verify if lithium microdose interfered with this activity, animals were submitted to mobility tests at 6 and 18 months of age. Considering locomotion, no diference was observed between Control WT or TG animals at both 6 and 18 months of age (Fig 2A). Treatment of TG mice with lithium did not change their locomotor activity as well (Fig 2B). It has been reported that transgenic animal models for Alzheimer disease present greater motoractivity in open field and object recognition tasks. This can be due to cortical and hippocampal atrophy in some of those animals [24,25,26]. However, this increase in locomotion was not verified in the present work. In WT groups, treatment with lithium did not influence locomotion (S1 Fig).

### 3. Evaluation of anxiety

**Fig 3:**
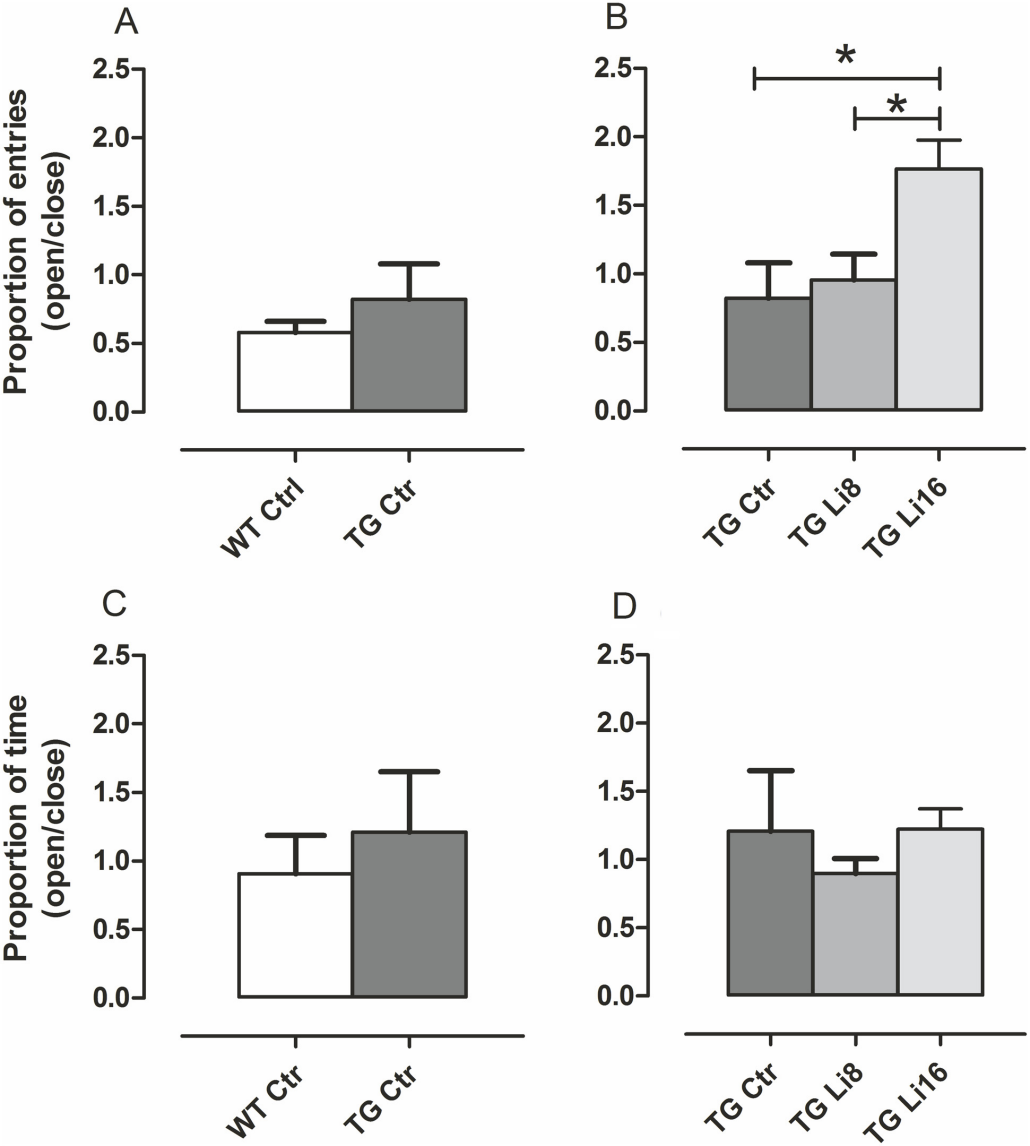
Lithium treatment reduces anxiolytic behavior in TG mice. Animals of both WT (n = 10) and TG (n = 5) strains were tested in Elevated Plus Maze when they completed 18 months old. Number of entries (A, C) and time spent (B,D) in closed and open arms were recorded and the ratio open/closed were plotted. There was no difference between WT and TG mice both in the proportion of entries and time spent in each arm (A,B). TG Li16 (n = 7) presented an increase in proportion of entries in relation to TG Ctrl and TG Li8 (n = 7) (C), although there was no difference among the groups for the ratio of time spent in open/closed arms (D). Data are plotted as mean ± SEM of ratios. *: P < 0.05

Anxiety, a common disorder observed in AD patients [27], was evaluated to verify if the lithium treatment could alter this behavior. Elevated plus maze is a test whose foundation is based on the conflict between the natural tendency of rodents to explore a novel environment versus the natural tendency to avoid narrow elevated open areas [28]. In our experiments, we measured this behavior as the ratio of the number of entries in open arms over the number of entries in closed arms and also the ratio of time spent in open arms over time spent in closed arms. There was no difference in both parameters when TG Ctrl and WT Ctrl animals were compared (P = 0.2721 and P = 0.4915, respectively,Fig 3A and 3C) showing that initially, animals from both strains had the same behavior. Besides, TG animals treated for 16 months showed significant increase in the proportion of entries in open/closed arms (P < 0.05), indicating a rise in anxiolytic behavior, although no significant difference was observed in the ratio among time spent in both arms (Fig 3B and 3D). Treatment of WT group with lithium microdose for 16 months also increased the proportion of entries in open/closed arms (P < 0.05) but did not modify the time spent in both arms (S2 Fig), indicating that even in the absence of a neurodegenerative process, this treatment can reduce anxiety.

It was already reported that animal models for AD, including APPSwInd, present increased anxiety and locomotor activity [29,30]. In our experiments, however, these behaviours were not observed. Nevertheless, other works [31] showed that treatment with lithium was effective to control anxiety in rats. In line with this description, in the present work an increase in anxiolytic-like behavior was observed. This suggests that lithium microdose may turn the general behavior to a calmer state, which may contribute in a chronic treatment to the efficacy of the pharmacological strategy.

### 4. Evaluation of spatial memory

Spatial memory was evaluated using a Barnes maze, an appropriate apparatus to follow an animal’s behaviour along the aging process, as animals do not need to be exposed to water. In this way, stress level is lower and this method is being used as an alternative to Morris water maze [32].

Hippocampus and some areas of the brain cortex are recruited during spatial memory formation and recovery. In this way, mice and rats with hippocampal lesions present decreased performance in Barnes maze [33]. Besides, this is one of the most compromised areas in patients with Alzheimer’s disease [34].

**Fig 4:**
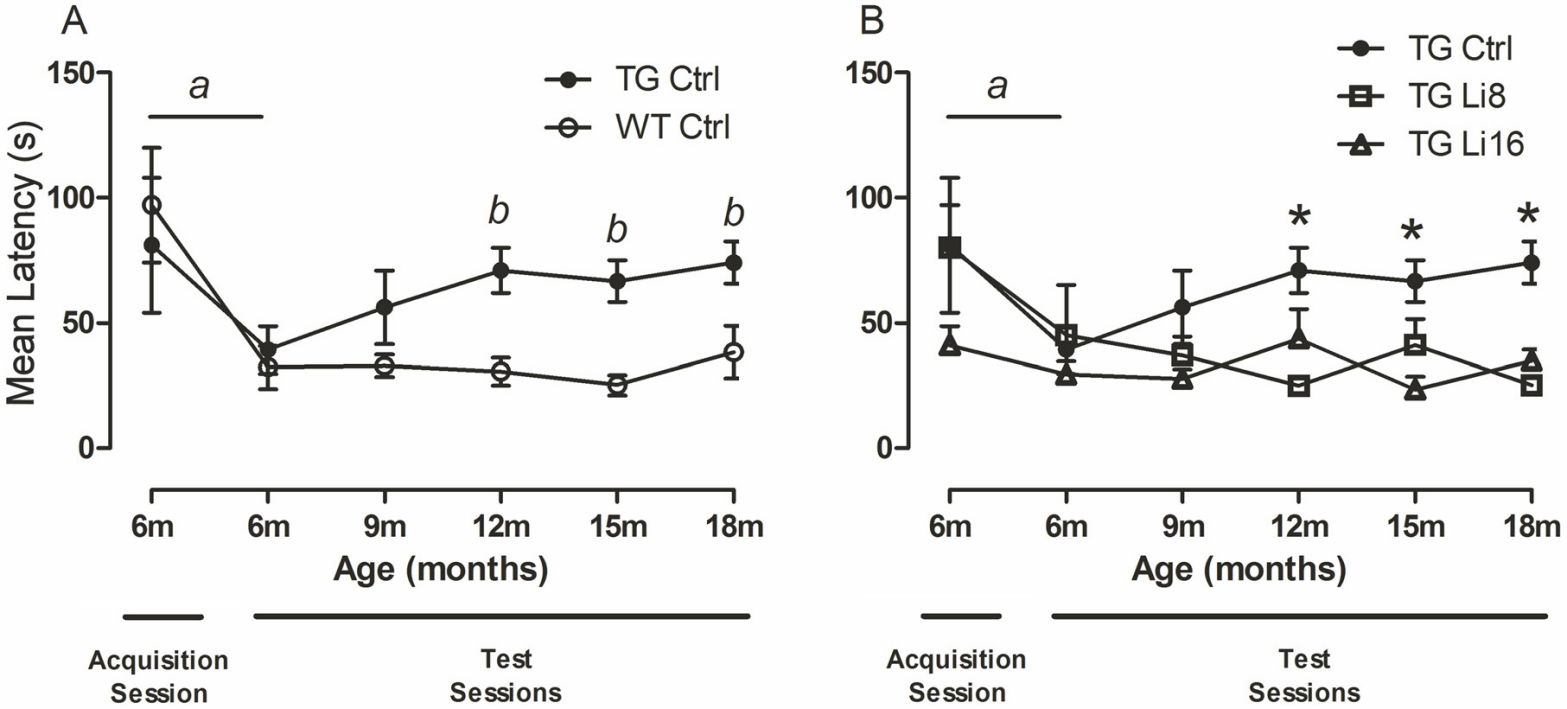
Lithium treatment prevents the loss of spatial memory in TG mice. Animals were first submitted to Barnes Maze when they were 6 months old, and we used a five-day protocol, exposing the animal twice a day to the task (Acquisition Session); latency was recorded. Both WT (n = 12) and TG Ctrl (n = 7) mice learned the task, as there is a reduction in latency to find the escape box in day 5 comparing to day 1 (A). Animals were re-exposed to the protocol every 3 months and TG Ctrl could not retain the memory, as its latency rose in test 2 (at 12 months of age) and kept higher than the latency of WT group up to the end of the experiments (A). We next compared the three TG groups and all of them learned the task, at 6 months of age (Acquisition Session). The treated groups, TG Li8 (n = 7) and TG Li16 (n = 8), did not present increased latency as did TG Ctrl, which means that the treatment with lithium was effective in preventing the loss of memory. Data plotted are mean ± SEM of latency. a: P<0.01, comparing days 1 and 5 within the groups; b: P < 0.01, WT Ctrl vs TG Ctrl. *: P < 0.05, TG Ctrl vs TG Li8 and TG Li16.

The animals’s first exposure to the maze was considered the “acquisition session”. Animals were submitted to the equipment twice a day, for 5 days, when they were 6 months old. All animals, regardless the strain, presented a significant reduction in latency to find the escape box [F_(1,129)_ = 8.22, P < 0.01) concerning the first (WT: 99.1 ± 24.8 s; TG: 81.0 ± 26.9 s) and the 5^th^ days (WT: 32.7 ± 9.2 s; TG: 39.2 ± 9.4 s), showing that all animals, at that age, had the capacity to learn the task. (Fig 3A). In the following months, spatial memory was evaluated every 3 months using the same protocol. TG Ctrl showed significant higher latencies to find the escape box when compared to WT Ctrl [F_(1, 131)_ = 8.22, P < 0.01], suggesting to have worse spatial memory. (Fig 4A). At 9 months, no difference in latencies of WT Ctrl or TG Ctrl animals was observed, neither among TG Ctrl nor TG treated animals. However, at later time points, TG animals treated with lithium for 8 or 16 months maintained the spatial memory of the maze, as their latency to find the escape box was significantly lower than TG Ctrl [F_(2, 156)_ = 10.47, P < 0.0001) (Fig 4B). It is important to point out that the improvement in behavior of TG Li16 can be due to improvements in the learning process, since those animals began the treatment with lithium four months before the training session. On the other side, the observed inprovement in TG Li8 may be related to better consolidation or retrieve of memory, since they learned the task at 6 months of age and the treatment began when they were 10 months old. This is remarkable information, as lithium microdose can be efficient to improve learning, memory consolidation and information retrieval. In the WT group, animals from both treatment times with lithium maintained spatial memory along the observed period (S3 Fig).

**Fig 5:**
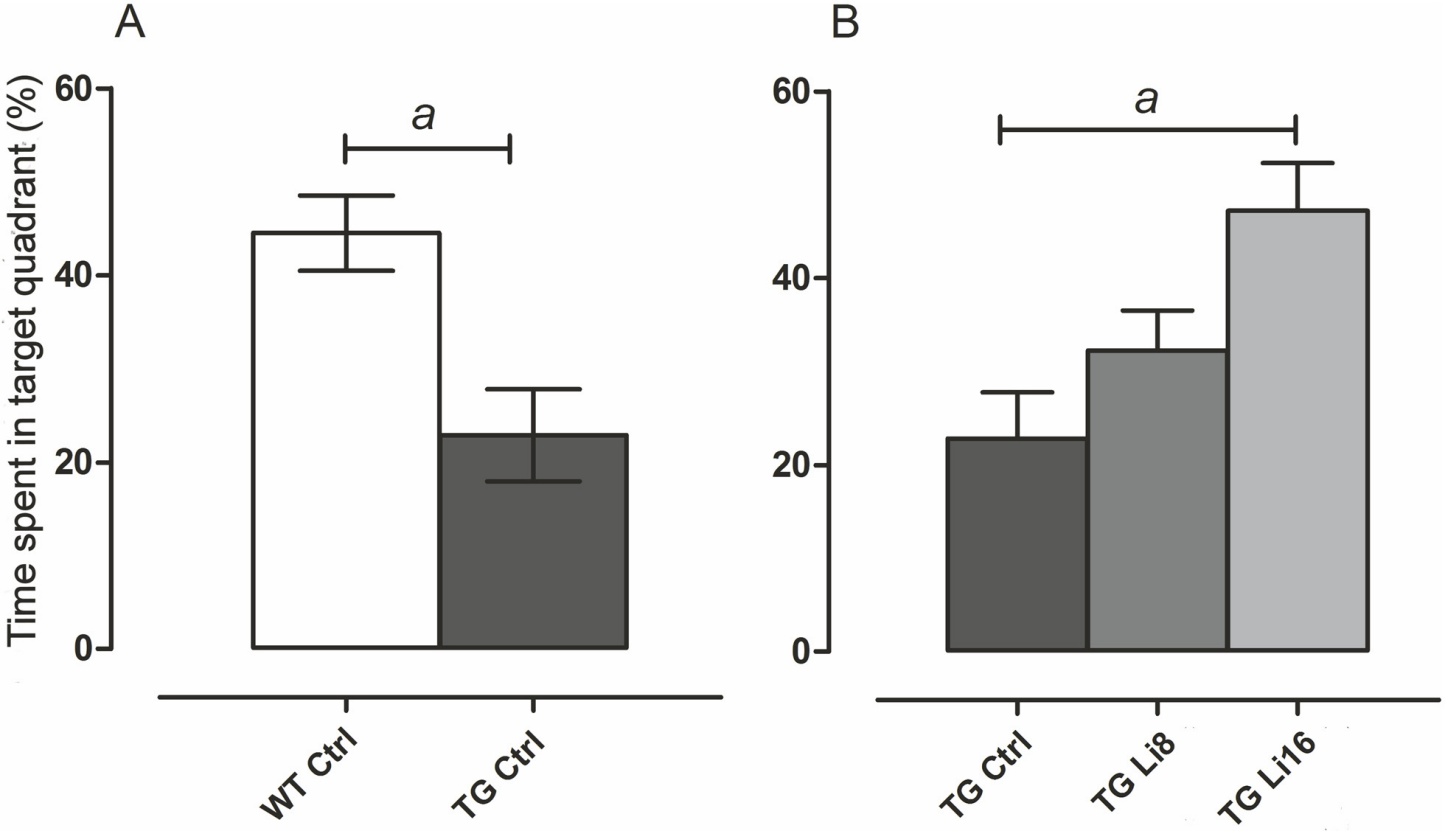
Lithium treatment increased the time spent in the target quadrant in Barnes maze. At 18 months of age the time spent in the target quadrant during the last Barnes maze test was evaluated. Control TG animals spent less time in the quadrant where the escape box was located. However, after the treatment with lithium for 8 or 16 months, this time was significantly increased indicating that those mice could remember where the escape box was located (*a*: P < 0.01).

In addition, distance covered to find the escape box and time spent in the target quadrant was evaluated in the last test, when animals were 18 months old. There was no difference in the distance covered by all groups. Nevertheless, regarding the time spent in the target quadrant, it was observed that TG Ctrl animals spent significant less time in the target quadrant (22.8 ± 4.9 s, P < 0.01) when compared to WT Ctrl animals (44.5 ± 4.0 s) (Fig 5A). However, the treatment with lithium for 8 or 16 months increased that time, a significant difference being observed in animals treated for 16 months (52.3 ± 6.8 s, P < 0.001), when compared to TG Ctrl animals (Fig 5B). There was no difference in this behavior concerning WT animals treated with lithium (S4 Fig).

So, treatment with lithium since 2 months of age until the elderly was effective in maintaining spatial memory of mice with or without neurodegeneration.

### 6. Evaluation of aversive-related memory

Aversive-related memory was evaluated in inhibitory avoidance equipment when animals were 18 months old. This test depends on the amygdala and the hippocampus, which is also a brain area affected in Alzheimer’s disease [35], bringing consequences for declarative and non-declarative memories.

**Fig 6:**
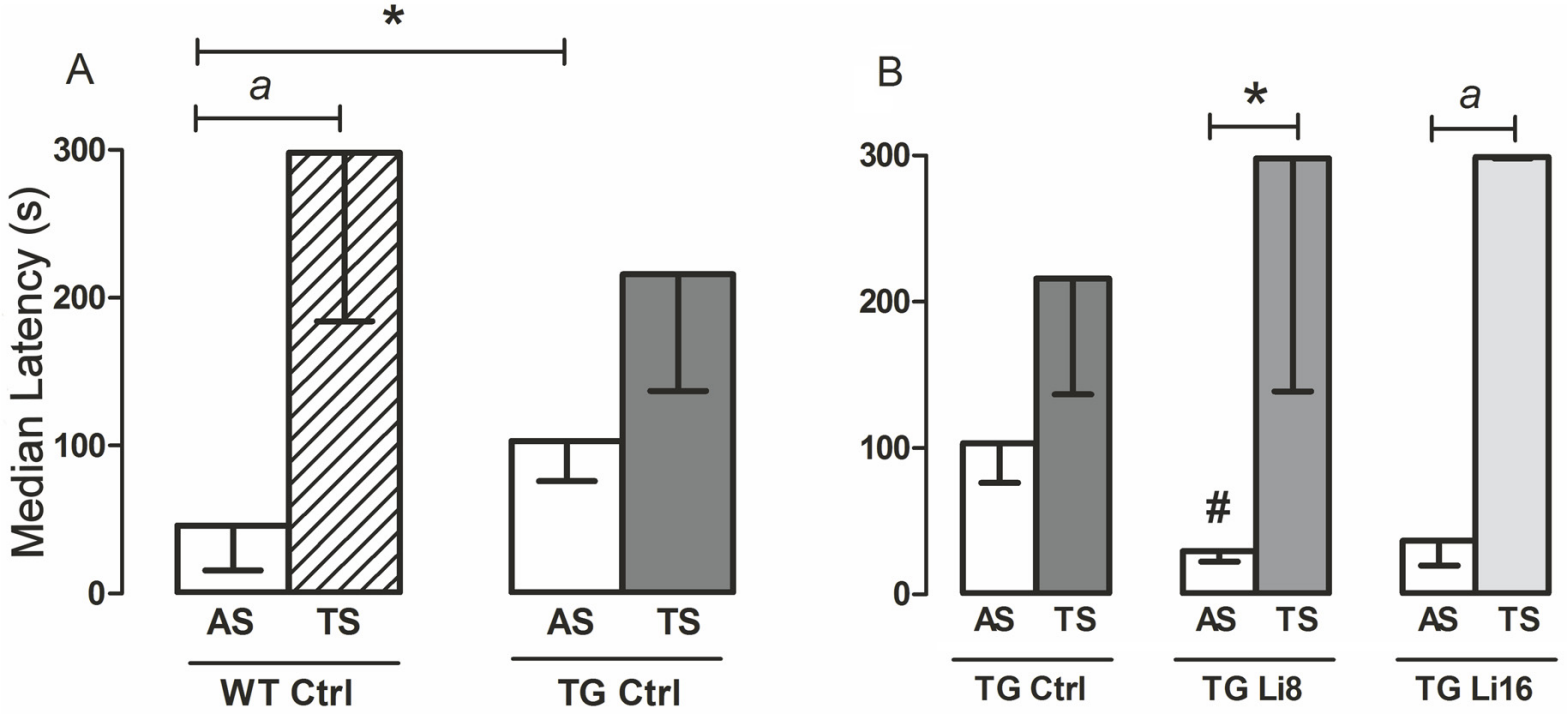
Lithium treatment protected TG mice from losing aversive-related memory. This type of memory was evaluated using an inhibitory avoidance apparatus in which animals were placed in a light box with access to a dark one, where they received a shock on the paws. The latency (max. 300s) to pass to the dark side was registered. Test session (TS) was performed 24 hours after acquisition session (AS). TG Ctrl (n = 7) showed higher latency in AS than WT (n = 10), and only the latter could remember the task, not passing to the dark side (A). Lithium reverted this result in TG Li8 (n = 8) and TG Li16 (n = 9), as the latency in TS of these groups was higher than in AS. Data plotted are median and interquartile range. *: P < 0.05, *a*: P < 0.01, #: P < 0.05 in relation to TG Ctrl “AS”.

Two sessions were performed (acquisition session–AS, and test session—TS), with a 24h time separating them. Initially, acquisition session of WT Ctrl and TG Ctrl were compared and it was verified that they were significantly different. Latency of TG Ctrl [103.30 s (73.40/134.30) s] was significantly higher than WT Ctrl [46.20 s (15.98/69.23) s, P < 0.05] (Fig 6A). Treatment with lithium for 8 or 16 months significantly reduced the time to enter the dark side in AS [29.8 s (22.7/40.0 s and 36.6 (19.8/42.4) s, respectively], when compared to TG Ctrl AS (Fig 6B).

In test session, it was observed that WT Ctrl animals were clearly able to remember the task, since latency to enter the dark box was significantly higher in the TS [298.0 s (184.0/298.0 s) when compared to AS [46.2 s (16.0/69.2 s), P < 0.01]. However, among TG Ctrl animals there was no significant difference between TS [216.0 s (137.0/298.0 s)] and AS medians [103.3 s (76.4/174.3 s)] (P = 0.1563, Fig 6A). Although some mice may remember the task (and maybe that is the reason why TS from WT Ctrl and TG Ctrl were not statistically different), the majority did not recognize and did not remember that the dark side of the shuttle box as aversive.. Treatment with lithium for 8 or 16 months significantly increased the latency to enter the dark side of the box by 7.9 times and 6.3 times [298.0 s (139.0/298.0 s, P < 0.05 and 299 s (298/300 s, P < 0.01)] respectively, when compared to latency in AS [29.8 s (22.7/40.0 s and 36.6 (19.8/42.4) s], respectively (Fig 6B). With these observations, it is possible to suggest that lithium treatment in microdose protects different types of memory processing from degradation. Besides, WT groups treated with lithium for 8 or 16 months also had their memory maintained along the aging process (P < 0.01 and P< 0.001, respectively) (S5 Fig). As mentioned before, our research team has already shown that the use of this microdose prevented memory deficit in human subjects. However, the effects were evident after a minimum of three months treatment [5]. This is probably due to some important brain adaptations that occur during the healing (or protection) process in the presence of lithium, as decribed next.

### 7. Histopathological analysis

Disruption of memory decay along the aging process of this murine model of Alzheimer’s disease can be related to promotion of neuroplasticity in brain areas related to cognitive domains. Neuroplasticity involves structural changes that may lead to functional improvements, which can explain the behavioral changes observed with the lithium treatment.

**Fig 7:**
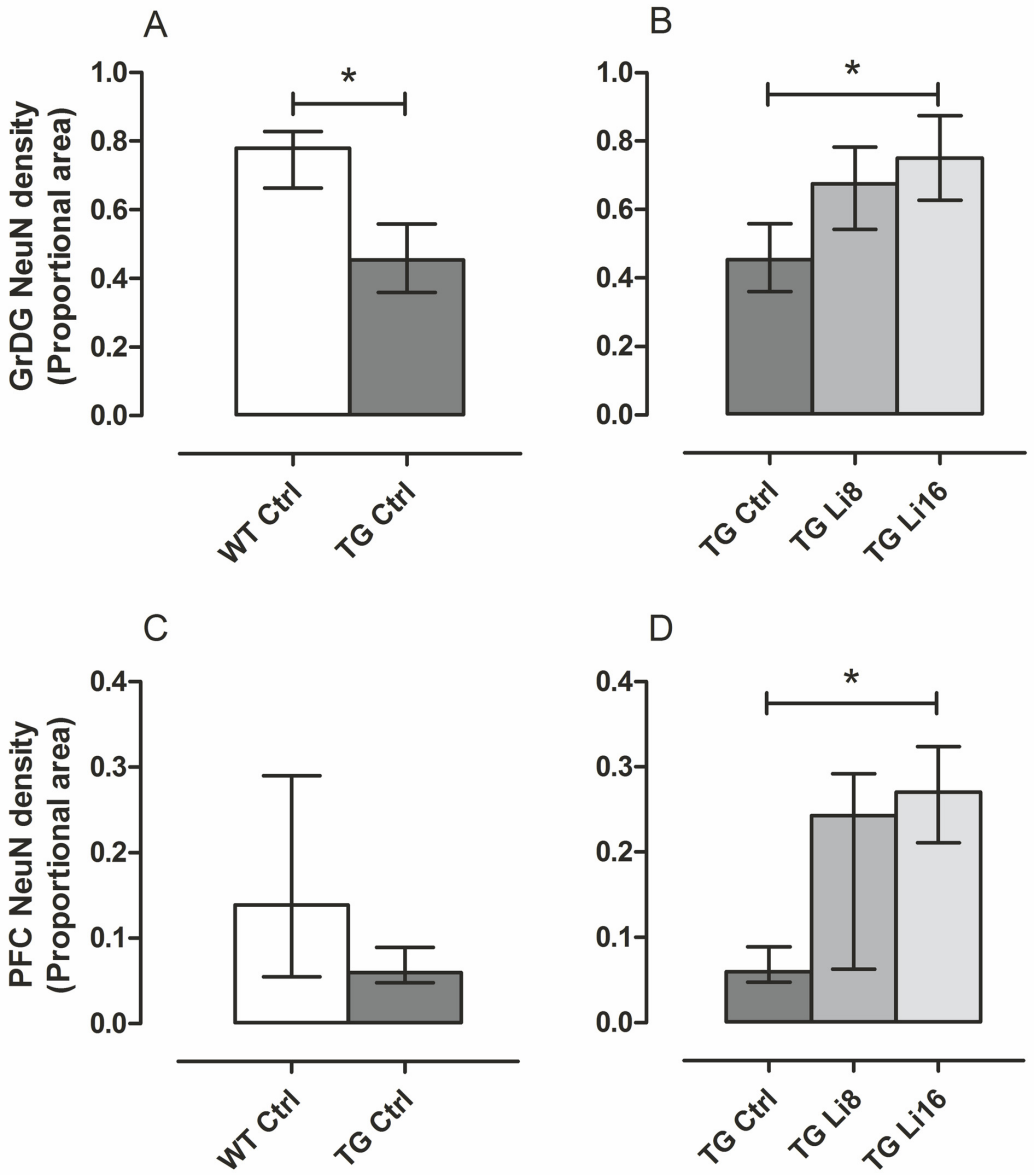
Lithium treatment prevented neuronal loss in TG mice treated for 16 months in GrDG and increased the density of neurons in prefrontal cortex. Brains sections were immunolabeled with NeuN antibody. TG Ctrl (n = 5) group had a decrease in proportional area stained compared to WT Ctrl (n = 4) (A). Treatment with lithium for 16 months, however, prevented TG Li16 group (n = 5) from this neuronal loss (B). The treatment for 8 months was not enough to produce the same effect, as proportional area stained in TG Li8 (n = 5) was not statistically different from TG Ctrl. In prefrontal cortex (PFC), there was no difference between WT and TG (C), but treatment with lithium resulted in higher proportional area stained in TG Li16, compared to TG Ctrl (D). Data plotted are median and interquartile range. *: P < 0.05.

**Fig 8:**
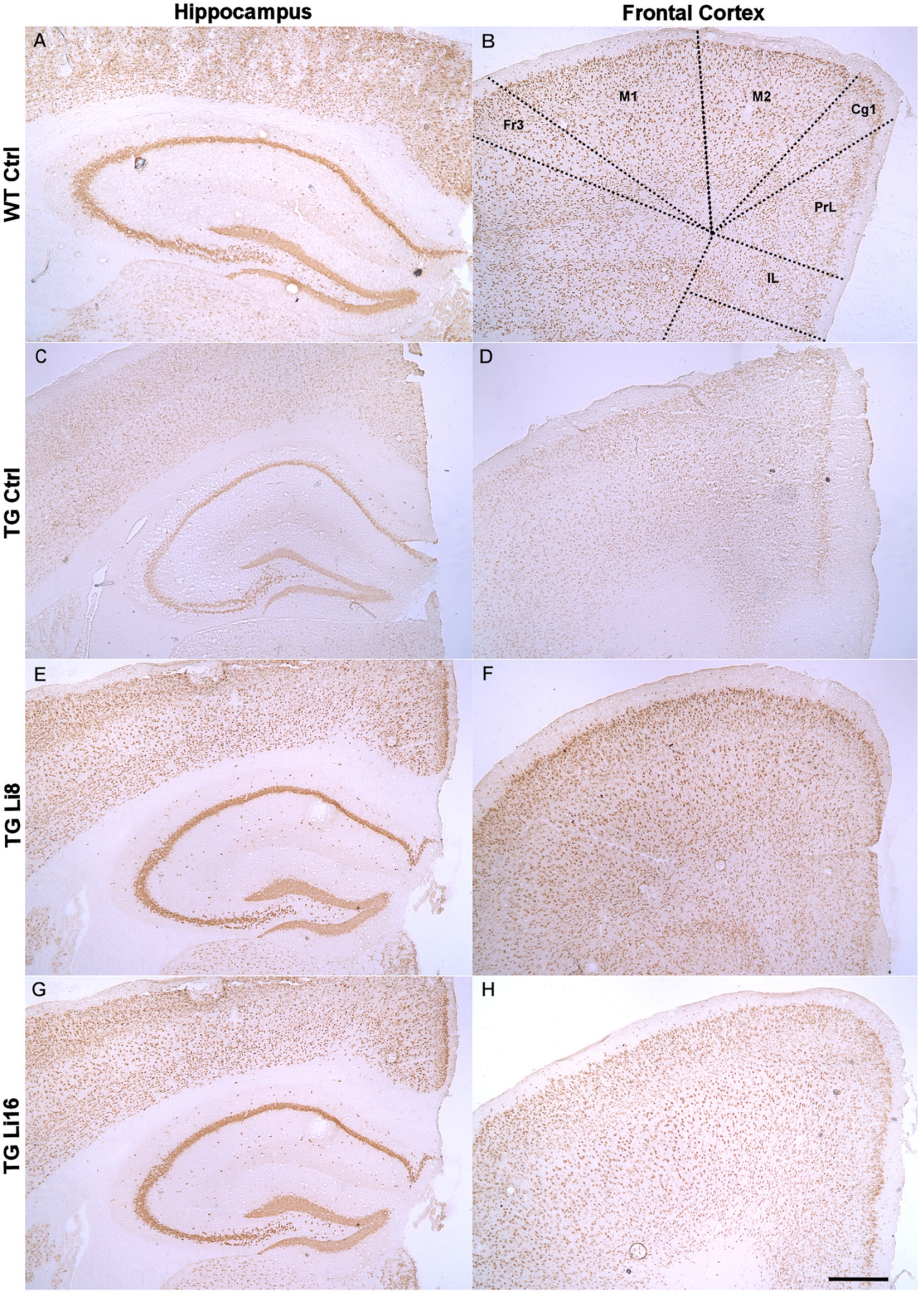
Representative figures of anti-NeuN immunoreactivity in frontal cortex and hippocampus of WT Ctrl (A and B), TG Ctrl (C and D), TG Li8 (E and F) and TGLi16 (G and H) groups. The coronal sections shown are at approximately the same anatomical level (left panels 1.98 mm and right panels –1.58 from Bregma). Representative images are from samples run in the same batch during immunohistochemistry procedure. Images in each row belong to the same animal and match with the animals showed on panels of thioflavine-S staining figure. Figure abbreviations for cortical areas: Fr3, frontal cortex, area3; M1, primary motor cortex; M2, secondary motor cortex; Cg1, Cingulate cortex, area1; PrL, prelimbic cortex; IL, infralimbic cortex. Anatomical localization and abbreviations of hippocampal structures are described in the line drawing in “panel B” of thioflavine-S staining figure. Scale bar is 400 μm.

In this way, the density of neurons was evaluated in the hippocampus and prefrontal cortex using an antibody for NeuN protein. In some hippocampal areas such as CA1, CA2 and CA3 and the polymorphic layer of dentate gyrus (PoDG) no difference between WT Ctrl and TG Ctrl samples was observed. In the same way, treatment with lithium did not change the density of NeuN in these areas (data not shown). However, in the granular layer of the dentate gyrus (GrDG), a significant reduction of 39.5% in NeuN labeling was observed in TG Ctrl animals, when compared to WT Ctrl samples (P < 0.05) suggesting a loss of cells in this area (Figs 7A and 8B and 8D). Treatment with lithium for 16 months significantly protected brains from this loss (P < 0.05), maintaining the density similar to WT Ctrl group (Figs 7B and 8F and 8H). In WT groups treated with lithium no difference in NeuN density in hippocampus or cortex was observed (S6 Fig).

These observations are in line with others that already described that lithium increases neurogenesis in the sub-granular zone of GrDG and prevents hippocampal apoptosis [36–37]. This can be related to the improved performance of animals from TG Li16 group in Barnes’ maze that presented better memory than TG Ctrl group. The hippocampus is highly recruited in this task and GrDG is involved in memory events that can be predicted by the animal [38–39] in order to navigate in a place that rescues spatial memory. For this, GrDG cells receive inputs from the entorhinal cortex perforant pathway [40]. GrDG acts like a learning net that withdraws input redundancies (sensorial inputs such as vestibular, olphactory, visual, audictive and somatosensorial) leading to more categorized outputs to be used in CA3 [41]. In this way, one can solve separation patterns of spatial clues [42]. GrDG plays an important role in data codification, helping to build spatial representations for posterior CA3 area.

Preservation in neuronal density in GrDG can also be associated with the anxiolytic effect observed in mice treated with lithium in the elevated plus maze test. Inputs to GrDG can reduce anxiety without affecting the learning process [43].

In the prefrontal cortex TG Ctrl mice displayed lower NeuN density than WT Ctrl animals, but this did not reach statistical significance. (Figs 7C and 8A and 8C). While no significant change in NeuN labeling was observed in TG Li8 samples (Figs 7D and 8E), treatment with lithium for 16 months significantly increased the density of neurons by 4 times (P < 0.05), when compared to TG Ctrl samples (Figs 7D and 8G), meaning that this treatment was able to protect the animal from the neuronal loss that was imposed by the disease. The prefrontal cortex is endowed with the expression of remote memories and the cingulate cortex, specifically, is directly related to the formation and retrieval of remote spatial memory [44,45] and with the hippocampus coordinated spatial memory [46]. During aging, neuronal activity is spread in the prefrontal cortex of both hemispheres as a system of compensation against neurodegenerative changes that may occur along the process [47]. Also, prefrontal region interacts functionally with both the ventral and dorsal brain. This area is being implicated as a neural center for attention driven by stimulus and objectives [48].

Besides the neuronal density, the preservation of synaptic terminals was also verified using an antibody against synaptophysin (p38) which is a transmembrane glycoprotein located in pre-synaptic vesicles [49]. A decrease in synaptophysin density was described earlier in this murine model of neurodegeneration [12], however, in the present study no difference between WT Ctrl or TG Ctrl animals was observed (S7 Fig). In the same way, the treatment with lithium made no difference in the density of this protein. This phenomenon was observed earlier by our group [50] and others where no significant difference in synaptophysin western-blotting between APP23 mice and wild-type controls were verified at 3 and 25 months of age [51] and could be explained by the enlargement of synaptic size to compensate de decrease in synapse number [52,53]. Also, the abscense of synaptophysin decrease could be related to a recognized trophic effect of APP contributing to a compensatory mechanism preventing or delaying the synaptic loss in face of the neuronal loss [54].

Still focusing on neuroplasticity, brain-derived neurotrophic fator (BDNF) was quantified in total hippocampus and cortex. This protein is involved in synaptic changes that occur during long-term potentiation and, as a consequence, in long-term memory formation [55]. In Alzheimer’s disease animal models, there is a decrease in density of BDNF [56] and lithium in higher dosis increased BDNF in cortex and hippocampus [57]. In fact, increase in BDNF levels may happen in many brain regions, maybe related to increase in mRNA exon IV and activity of BDNF promoter IV [58].

**Fig 9:**
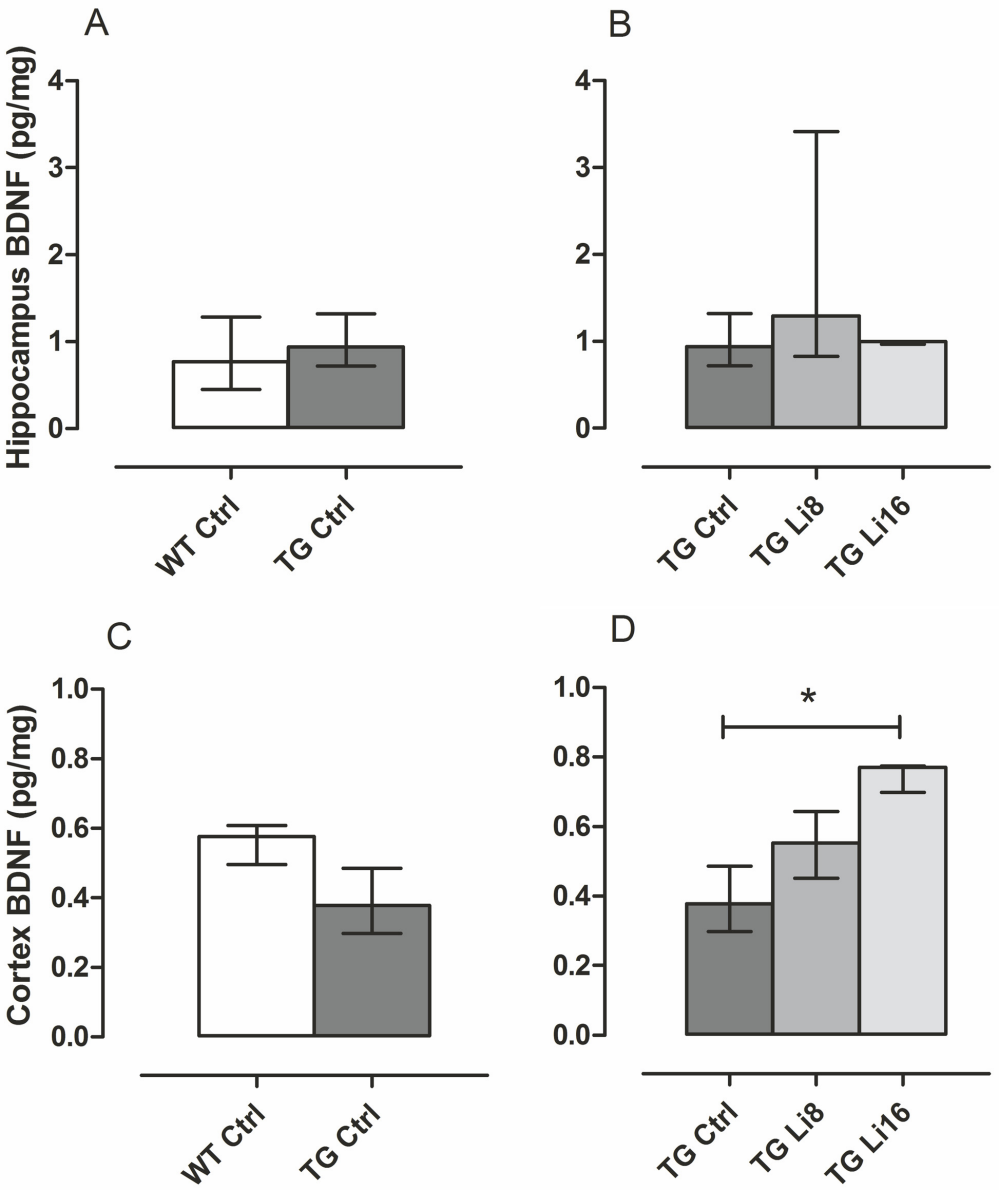
Lithium treatment for 16 months increased BDNF levels in cortex of TG mice. Brain homogenates were tested to BDNF by ELISA. There was no difference in BDNF levels either in hippocampus (A) or in cortex (C) of TG Ctrl (n = 3) when compared to WT Ctrl (n = 3). In the same way, lithium treatment did not influence in BDNF levels in hippocampus of TG mice (B). In cortex, however, TG Li16 (n = 3) presented higher levels of the neurotrophic factor, compared to TG Ctrl. Although TG Li8 has presented a slight increase in BDNF, it was not significant. Data plotted are median and interquartile range. *: P < 0.05.

In the present study, although the analysis of BDNF density in hippocampus and cortex were performed with only three samples of each group, some preliminary and exciting findings can be described. In the hippocampus there was no difference in total BDNF density of TG Ctrl animals, when compared to WT Ctrl (Fig 9A and 9C). In the same way, no difference in hippocampus was observed with the treatment of animals with lithium (Fig 9B). However, in the cortex, as observed with NeuN labelling, TG Ctrl group presented lower BDNF density than WT Ctrl, with no statistical difference. Nervertheless, treatment of TG mice with microdose lithium for 16 months significantly increased (P < 0.05) by 2.5 times the density of this neurotrophin in cortex, when compared to the density of TG Ctrl mice [0.38 (0.30, 0.49) pg/mg protein, Fig 9D]. Probably the maintenance of neuronal bodies in cortex was promoted by the increase in neurogenesis, with increase in BDNF.

Treatment of WT animals with lithium did not alter BDNF density in the cortex or hippocampus (S8 Fig).

It was already reported that BDNF/TrkB signaling pathways are essential to normal cortical neurogenesis [59]. This neurotrophin also stimulates activation of endothelial nitric oxide synthase (eNOS) which promotes NO increase in neural stem cells that reciprocally regulate BDNF expression. This mechanism was verified after cerebrovascular accident in mice [60].

In the hippocampus, the observed neuroprotection may be related to other molecular mechanisms [61], but BDNF. One probable molecular pathway for the neuroprotection promoted by lithium chronic treatment is the protection against excitoxicity induced by glutamate mediated by NMDA receptors, as lithium can inhibit calcium influx through NMDA receptors [62,63]. Also, it was already described that lithium treatment can induce survival molecules in the frontal cortex, such as Bcl-2, an anti-apoptotic protein that inhibits the release of cytochrome-C from mitochondria, regulating permeability of the external mitochondrial membrane [64,65]. Besides, lithium can also keep homeostasis in endoplasmatic reticulum and induce neurogenesis by inhibition of GSK-3 protein and activation of ERK cascades [66,67].

**Fig 10:**
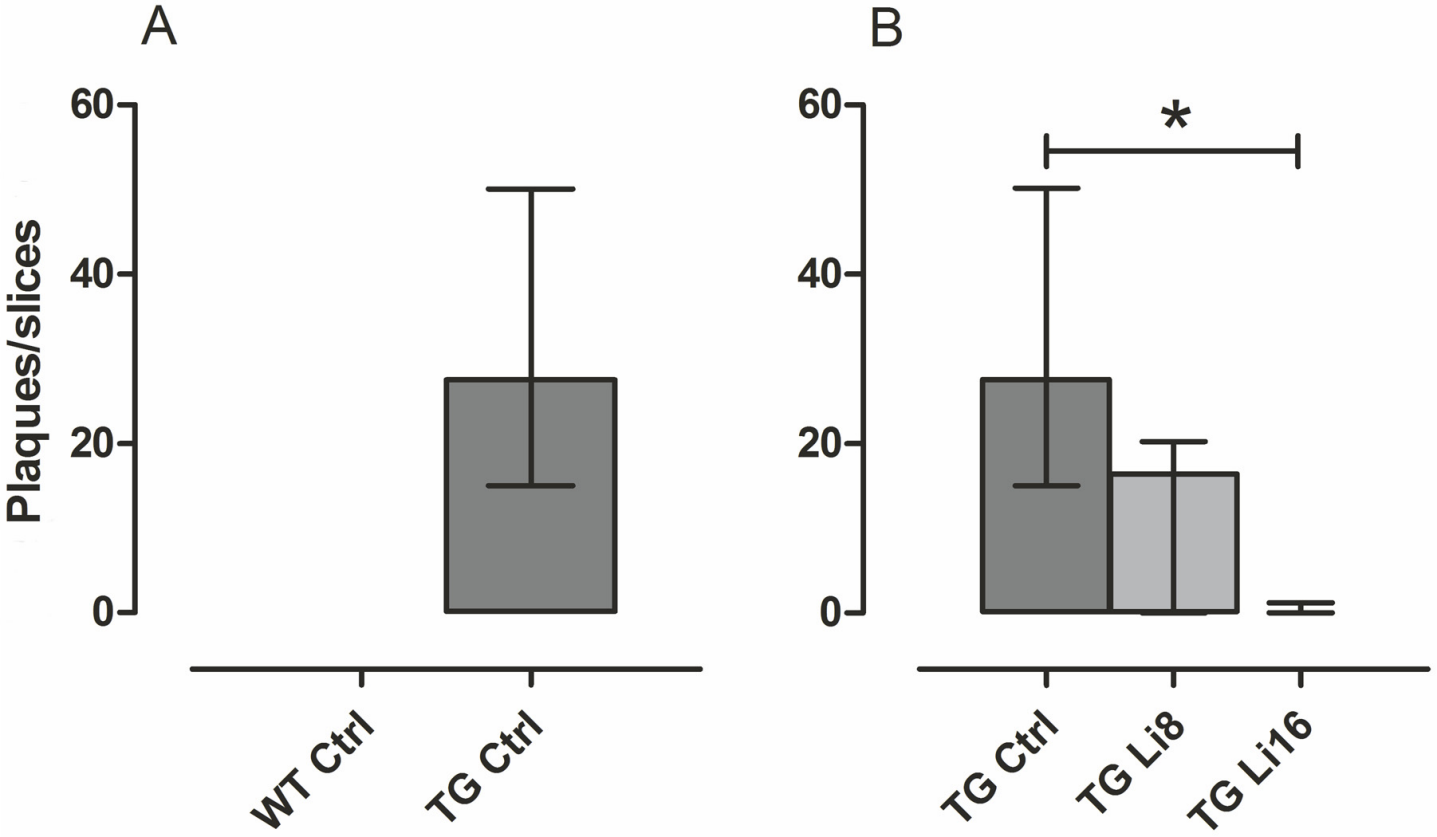
Lithium treatment reduced the number of amyloid plaques in TG mice. Brains sections were stained with Thioflavine-S and plaques were counted in the whole section. The ratio of plaques/slice is plotted. WT Ctrl (n = 4) did not present any plaques. Treatment with lithium during 16 months reduced the number of plaques in TG Li16 (n = 5) compared to TG Ctrl (n = 6). TG Li8 (n = 5) also presented a reduction but it was not statistically different. Data were plotted in median and interquartile range. *: P < 0.01.

**Fig 11:**
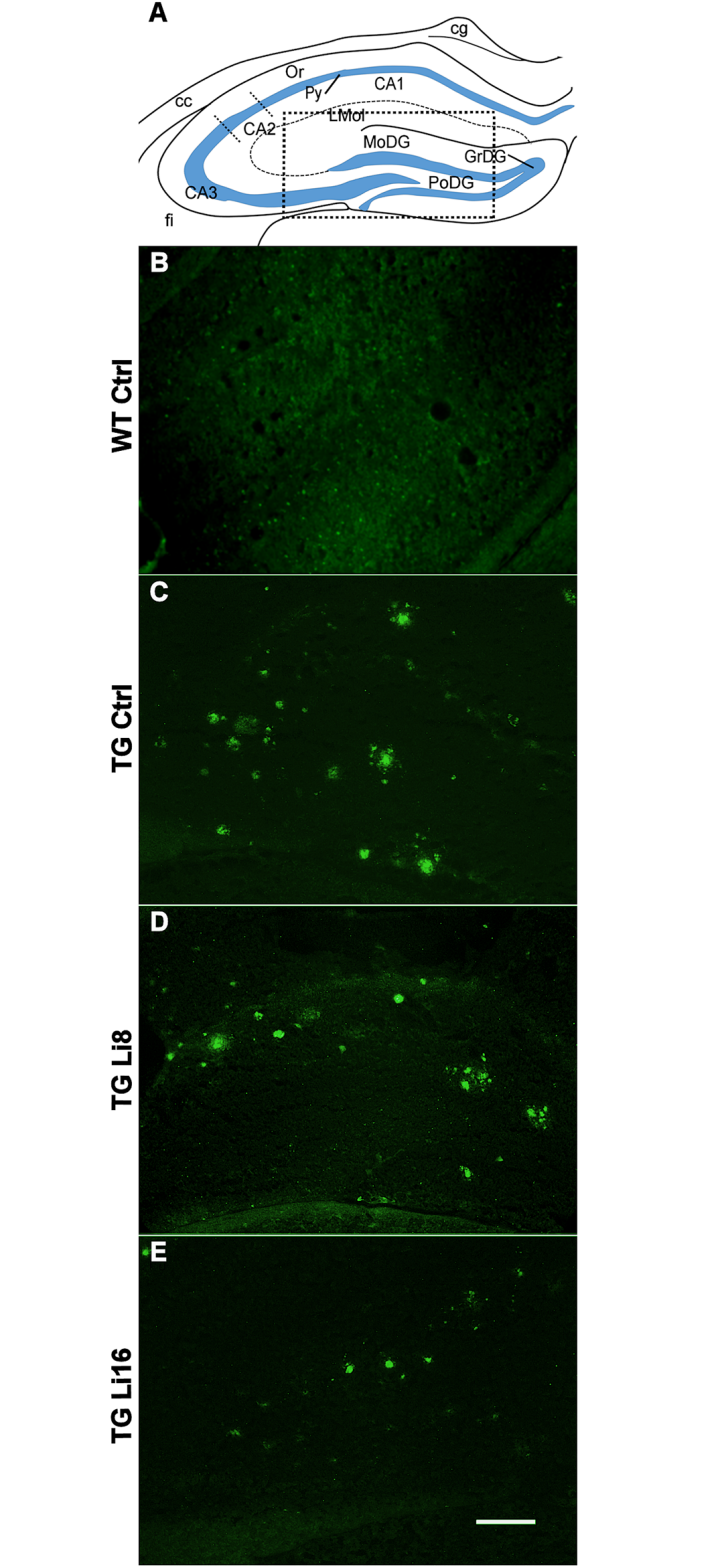
Representative figures of thioflavine-S staining in the hippocampus of WT Ctrl (B), TG Ctrl (C), TG Li8 (D) and TGLi16 (E) groups. The coronal sections shown are at approximately the same anatomical level (-1.58 from Bregma). Representative images are from samples run in the same batch during staining procedure. Images match with the animals showed on Fig 8. Hippocampal areas are described on line draw from “A” and the dotted square depicts the approximated area shown on panels “B” to “E”. Structure abreviations were cited according to Franklin and Paxinos, 2008 [69]. Figure Abbreviations: cg, cingulum; CA1, field CA1 of hippocampus; CA3, field CA3 of hippocampus; Lmol, stratum lacunosum moleculare of the hippocampus; CA2, field CA2 of hippocampus; Py, pyramidal cells of field CA1 of hippocampus; PoDG, polymorphic layer of the dentate gyrus; GrDG, granular layer of the dentate gyrus; MoDG, molecular layer of the dentate gyrus; fi, fimbria. Scale bar is 160 μm.

Neuroprotection was also verified determining the potential of the treatment with lithium microsode to inhibit the formation of senile plaques. Senile (or amyloid) plaques are one of the main characteristics of Alzheimer’s disease. They are composed of a central core with amyloid fibrils surrounded by dendrites, distrophic axon terminals and activated microglia [68]. In this work a great number of plaques were observed in the brain of transgenic animals [(27.55 (15.00, 50.15) plaques/slice/animal, Figs 10A and 11C and 11D]. The treatment with lithium for 8 months, decreased the number of plaques in TG mice, but it was not significant (Figs 10B and 11E and 11F). However, treatment for 16 months significantly reduced by 98% (P < 0.01) the density of plaques in TG animals, when compared to the control group (Figs 10B and 11G and 11H). These data reinforces the neuroprotective role of lithium against the characteristic neurodegeneration observed in Alzheimer’s disease.

Inhibition of plaque formation seemed to be essential to the observed behavioural alterations, as deposition of plaques in hippocampus has already been associated to changes in behavior of mice [70] and humans [71]. One of the most known pathways related to formation and pathogenicity of amyloid plaques is GSK3 pathway. The isoforms GSK-3α and GSK-3β are related to the neurodegenerative process of Alzheimer’s disease by its mechanisms of tau protein hyperphosphorylation and plaque formation stimulus involving induction of inflammatory cascades, increases in the production of amyloid-β peptide, among other pathways activated by this enzyme [72,73,74]. Although not tested in the present work, other hypothesis that can be involved with the molecular mechanism of amyloid plaques formation or degradation is the autophagy and the increase in oxidative stress. Autophagy is a process that is negatively regulated by mTOR and positively regulated by inhibition of inositol monophosphatase; both of them may be regulated by chronic treatment with lithium [75, 76]. Besides, it was already reported that the reduction in autophagy promotes neurodegeneration similar to Alzheimer’s disease in mice [77]. The oxidative stress, a mechanism directly associated to production of amyloid-β peptide [67] is another pathway that can be inhibited by lithium [78] and could be another explanation for the observed reduction of amyloid plaques.

In other to verify one of those possible mechanisms of neuroprotection, GSK-3β activity was studied in brain homogenates of transgenic mice. GSK-3 family was highly conserved along the evolution. In humans it is coded by two distinct genes: GSK-3α (51 kDa) and GSK-3β (47 kDa) [6]. This enzyme family has an important role in cellular survival and apoptosis [79]. Both isoforms are related to Alzheimer’s disease [80,74].

Lithium is the major inhibitor of these enzymes. It induces phosphorylation in inhibitory serine sites [80,81,82] and evaluation of the enzymes activity is critical for the understanding of lithium effects.

The activity of GSK-3β was quantified in the cortex and the hippocampus by the proportion of density of phosphorylated (inactive) form and the total density of the enzyme. With this microdose, no statistical difference was observed in this activity in both areas (S9 Fig). The lack of difference in pGSK3-β was already described in another hAPP transgenic mice model when compared to WT mice [8]. In that same work, a decrease in the activity of pGSK-3β after the treatment with lithium in a concentration 80 times higher than the dose we used (20 mg/kg/day/ versus 0.25 mg/kg/day) was described.

Despite the beneficial effect of GSK-3β inhibition for the decrease of Aβ deposition [8,9] and tau hyperphosphorylation [83], the decreased activity of this enzyme could lead to deleterious conditions in the brain, since GSK-3β regulates developmental processes, including neurogenesis, migration, axon growth and guidance, and synaptic plasticity [84]. Its activity is controlled through several signaling pathways activated by growth factors, wingless (Wnt) proteins, G-protein-coupled receptors (GPCR), β-arrestin, among others [85]. Also, GSK-3 plays an important role in various aspects of the regulation of glucose transport and metabolism. Chronic inhibition of GSK-3 enhanced glucose uptake and glucose transporter 1 (GLUT1) expression in TSC2-expressing (tuberous sclerosis complex 2) cells but not in cells lacking functional TSC2 [86]. In this way, the decrease in GSK-3 activity could lead to a pathological increase in intracellular concentration of glucose [87]. Our data showing the decrease in amyloid deposition due to lithium treatment, as well as memory and neuronal density preservation, and no significant alterations in pGSK-3β reinforces the relevance and safety of microdose lithium treatment. Than, the hypothesis that a long duration undetectable inhibition of GSK-3β could be enough to change the Aβ accumulation along the aging process should be considered.

Recently it was shown that long-term lithium treatment reduces glucose metabolism in the cerebellum and in the hippocampus of nondemented older adults. These alterations were not associated with any clinical evidence of toxicity and clinical implications need to be clarified [88]. It should be remarked that those authors used doses varying from 150 to 600 mg/day of lithium salt, which could implicate in metabolic alterations due to GSK-3β inhibition and cellular toxicity, as discussed above.

In the present work, considering the treatment duration, slight differences in the activity of this enzyme could happen and contribute to the general improvement in cerebral functioning in TG mice. More studies are needed to confirm this hypothesis. In WT animals treated with lithium, no difference in the GSK-3β activity was observed as well (S9 Fig).

## Conclusion

This work is the first to show that chronic microdose lithium treatment can prevent memory deficits caused by progressive neurodegeneration. In addition, it is the first to show that this treatment can alter the neurohistopathological characteristics of Alzheimer’s disease. In this way, the treatment did not alter the animals’ mobility, but drove transgenic mice to a less anxious state, which may contribute to the treatment along the aging process.

Protection of both aversive-related and spatial memories was observed, making their behavior similar to that observed in age-matched controls. This improvement was correlated to neuroprotection, as lithium protected the neuronal loss in the hippocampus and increased the density of neurons in the prefrontal cortex. This could be related to improvement in attention and memory retention along treatment.

Treatment with lithium also increased BDNF density in the cortex which accounts for advances in neuronal communication that is surelly degraded in neurodegenerative processes. This degration is also related to the presence of senile plaques. Plaques density was significantly reduced with the chronic treatment with lithium.

Lithium treatment did not promote behavioral or neurohistopathological alterations in WT animals suggesting that it could be used as a safe preventive pharmacological therapy, despite knowing if the individual will develop or not Alzheimer’s disease.

It is possible that microdose lithium has suppressed expression of the APP transgene in TG mice which would explain most of the results presented here. Although this hypothesis was not tested, it cannot be totally excluded.

Taking together, these data reinforce the protective effect of lithium already observed in human subjects. Moreover, they form an important basis to ensure that lithium microdose can alter the pathological characteristics of Alzheimer’s disease, leading to a new hope for the therapeutic treatment of the disease.

## Supporting Information

S1 Fig Treatment with lithium did not influence locomotion of WT animals. (TIF)

S2 Fig Treatment with lithium can reduce anxiety even in absence of neurodegeneration. (TIF)

S3 Fig WT animals trated with lithium maintained spatial memory. (TIF)

S4 Fig Lithium treatment did not influence the strategy to find the target in Barnes maze. (TIF)

S5 Fig Lithium treatment preserved the aversive-related memory of WT mice. (TIF)

S6 Fig Density of neurons was maintained in WT mice treated with lithium. (TIF)

S7 Fig Lithium treatment did not influence synaptic labeling. There was no difference in synaptophysin labeling between WT Ctrl and TG Ctrl animals. In the same way, lithium treatment did not influence synaptic density in both WT or TG groups.
(TIF)

S8 Fig Lithium treatment did not alter BDNF density of WT animals. (TIF)

S9 Fig No difference in GSK-3β activity was observed in the brain areas of treated mice. GSK-3β activity was measured by the proportion of density of phosphorylated (inactive) form and the total density of the enzyme. There was no difference in GSK-3β activity between WT Ctrl and TG Ctrl animals. In the same way, microdose lithium treatment did not change the GSK-3β activity in WT or TG mice.
(TIF)
