# Correction: Chronic Microdose Lithium Treatment Prevented Memory Loss and Neurohistopathological Changes in a Transgenic Mouse Model of Alzheimer's Disease

**DOI:** 10.1371/journal.pone.0145695

**Published:** 2015-12-17

**Authors:** Marielza Andrade Nunes, Natalia Mendes Schöwe, Karla Cristina Monteiro-Silva, Ticiana Baraldi-Tornisielo, Suzzanna Ingryd Gonçalves Souza, Janaina Balthazar, Marilia Silva Albuquerque, Ariadiny Lima Caetano, Tania Araujo Viel, Hudson Sousa Buck

There is an error within the fourth sentence of the Abstract. The correct sentence is: Transgenic mice (Cg-Tg(PDGFB-APPSwInd)20Lms/2J) and their non-transgenic litter mate genetic controls were treated with lithium carbonate (0.25mg/Kg/day in drinking water) for 16 or 8 months starting at two and ten months of age, respectively.

There is an error within the first sentence of the second paragraph of the Results and Discussion section. The correct sentence is: Lithium was administered as lithium carbonate in the dose 0.25 mg/Kg/day.
