# Supplementary figures and images for: Chronic Microdose Lithium Treatment Prevented Memory Loss and Neurohistopathological Changes in a Transgenic Mouse Model of Alzheimer's Disease
Source: PLoS One. 2015 Nov 25;10(11):e0142267. doi: 10.1371/journal.pone.0142267 (PMC4659557; doi:10.1371/journal.pone.0142267)

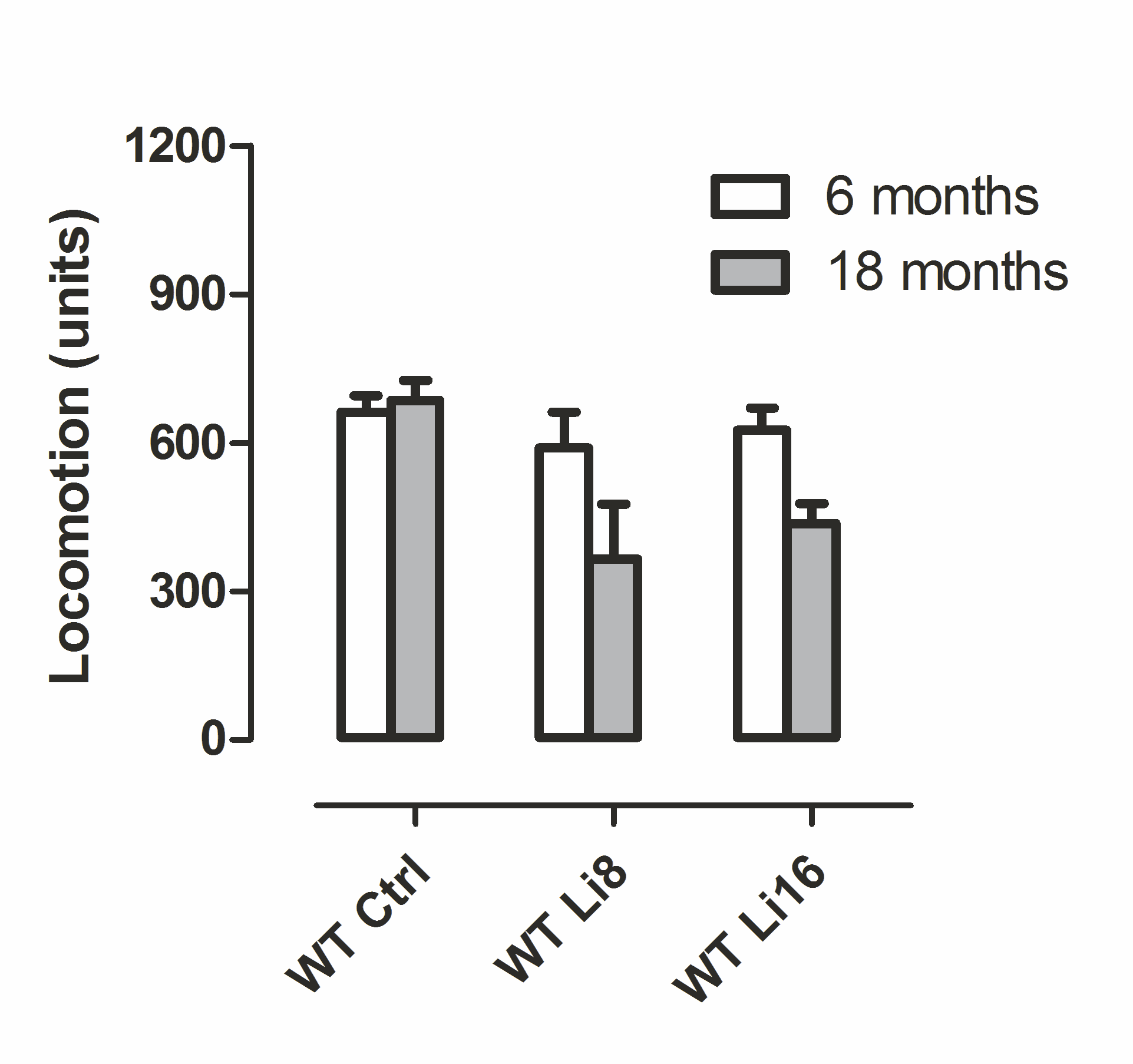

Supplement: S1 Fig — (TIF) [file pone.0142267.s001.tif]

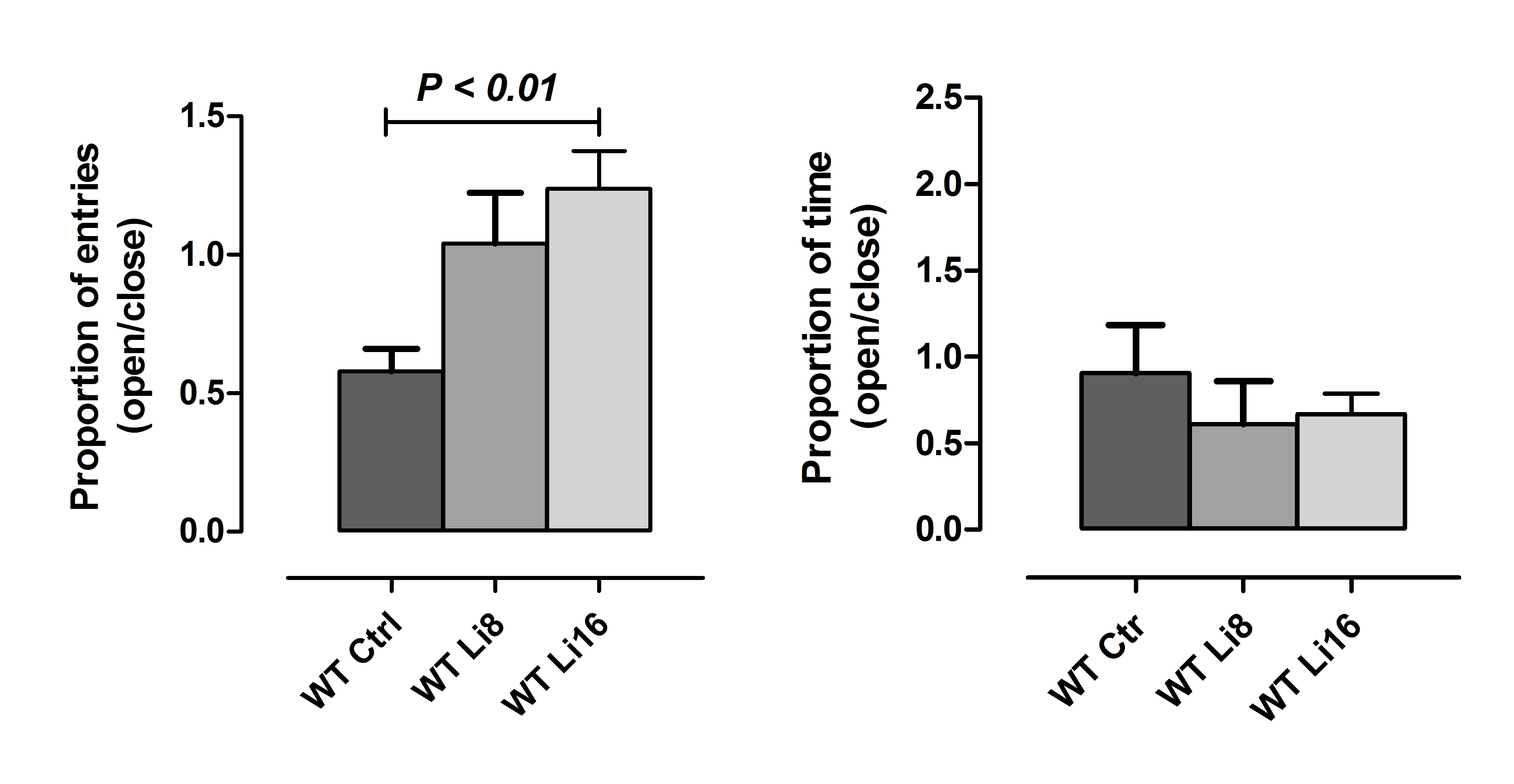

Supplement: S2 Fig — (TIF) [file pone.0142267.s002.tif]

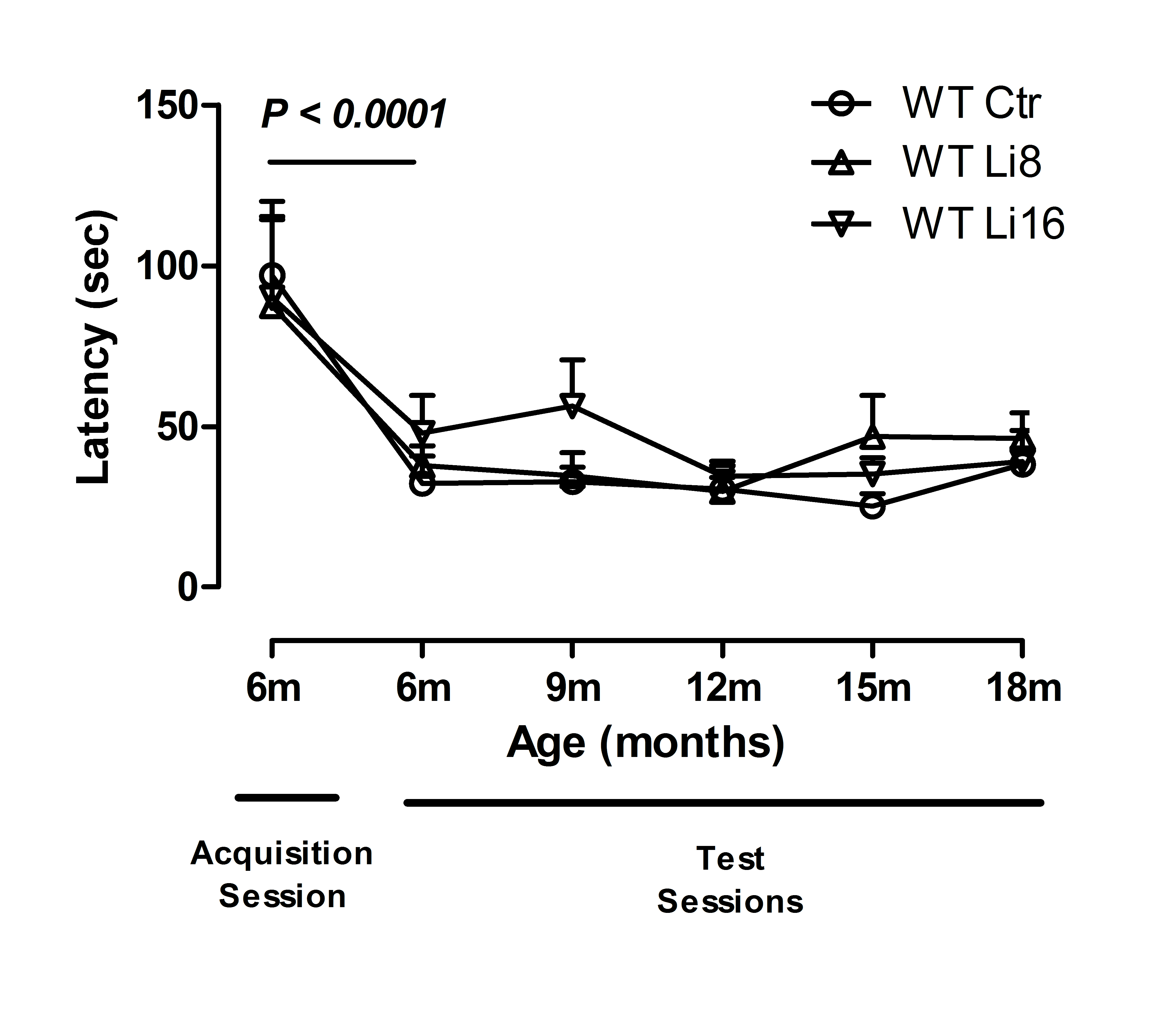

Supplement: S3 Fig — (TIF) [file pone.0142267.s003.tif]

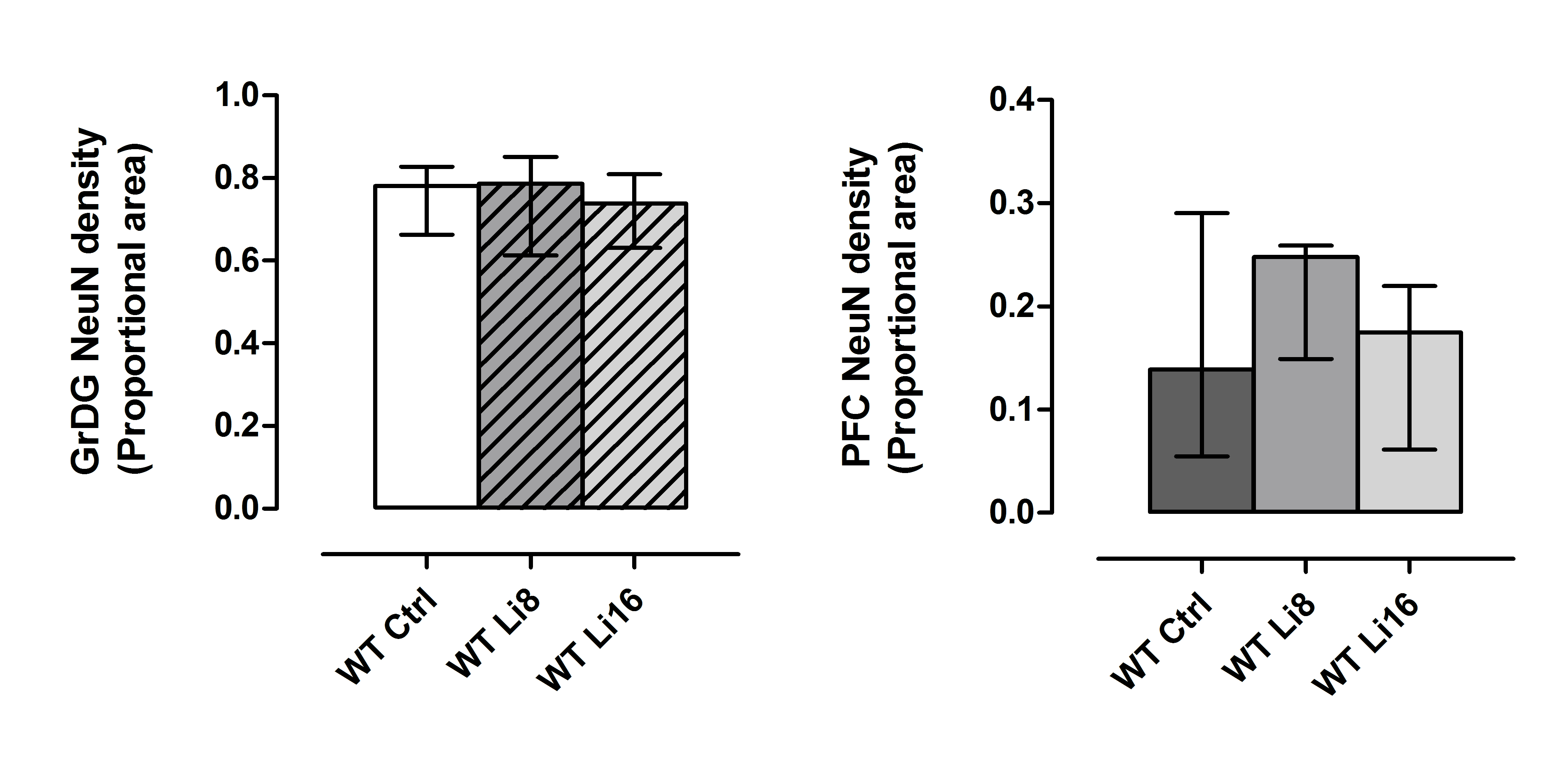

Supplement: S6 Fig — (TIF) [file pone.0142267.s006.tif]

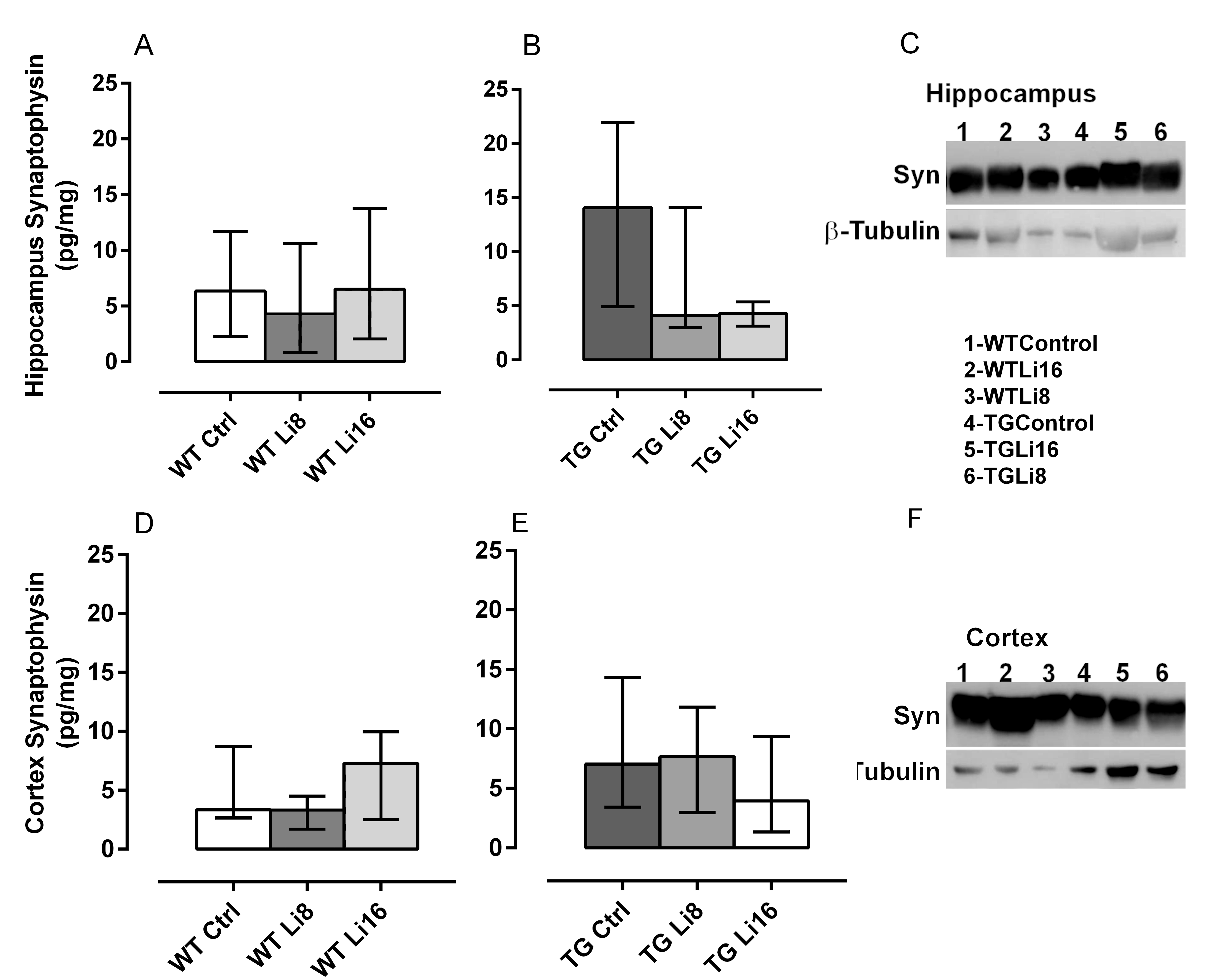

Supplement: S7 Fig — There was no difference in synaptophysin labeling between WT Ctrl and TG Ctrl animals. In the same way, lithium treatment did not influence synaptic density in both WT or TG groups. (TIF) [file pone.0142267.s007.tif]

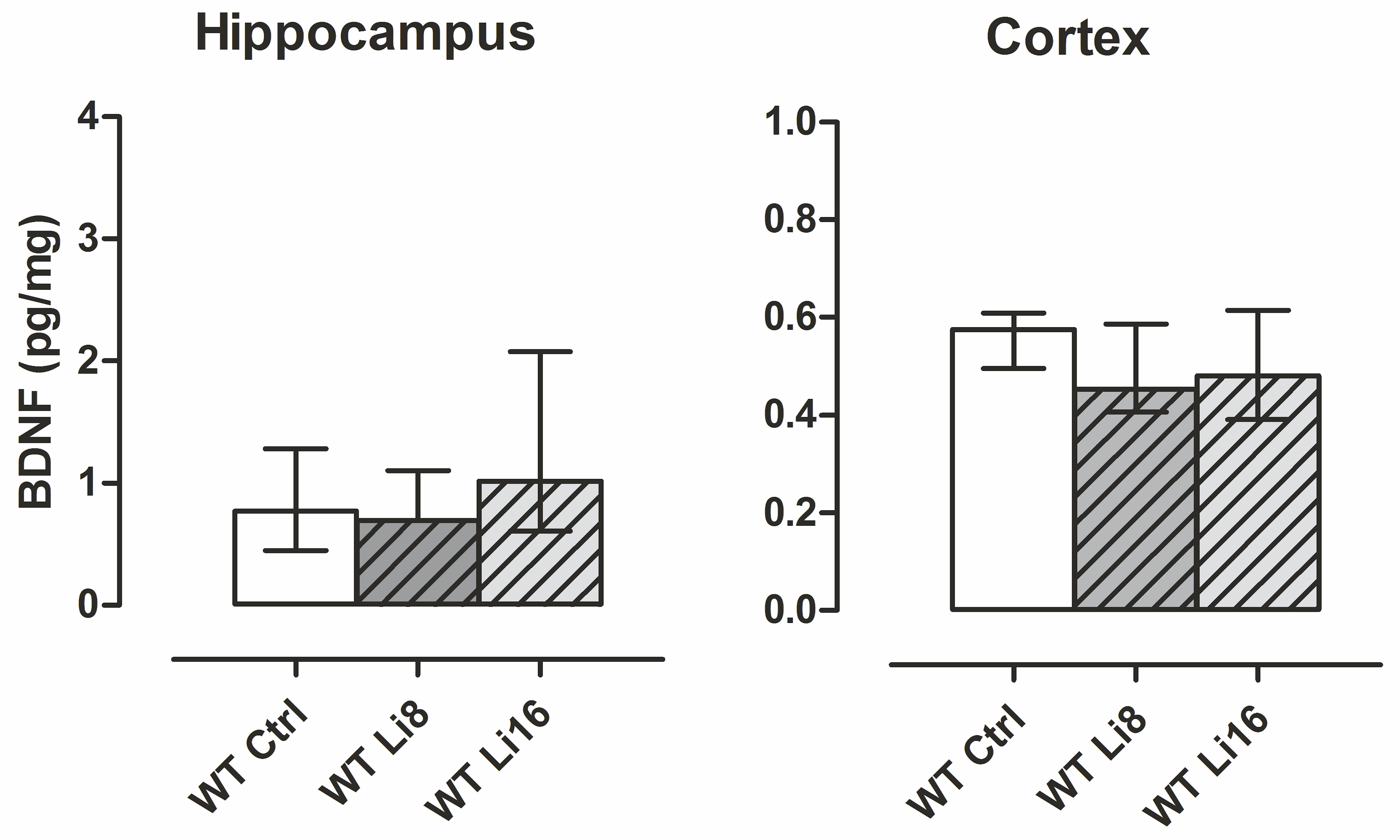

Supplement: S8 Fig — (TIF) [file pone.0142267.s008.tif]

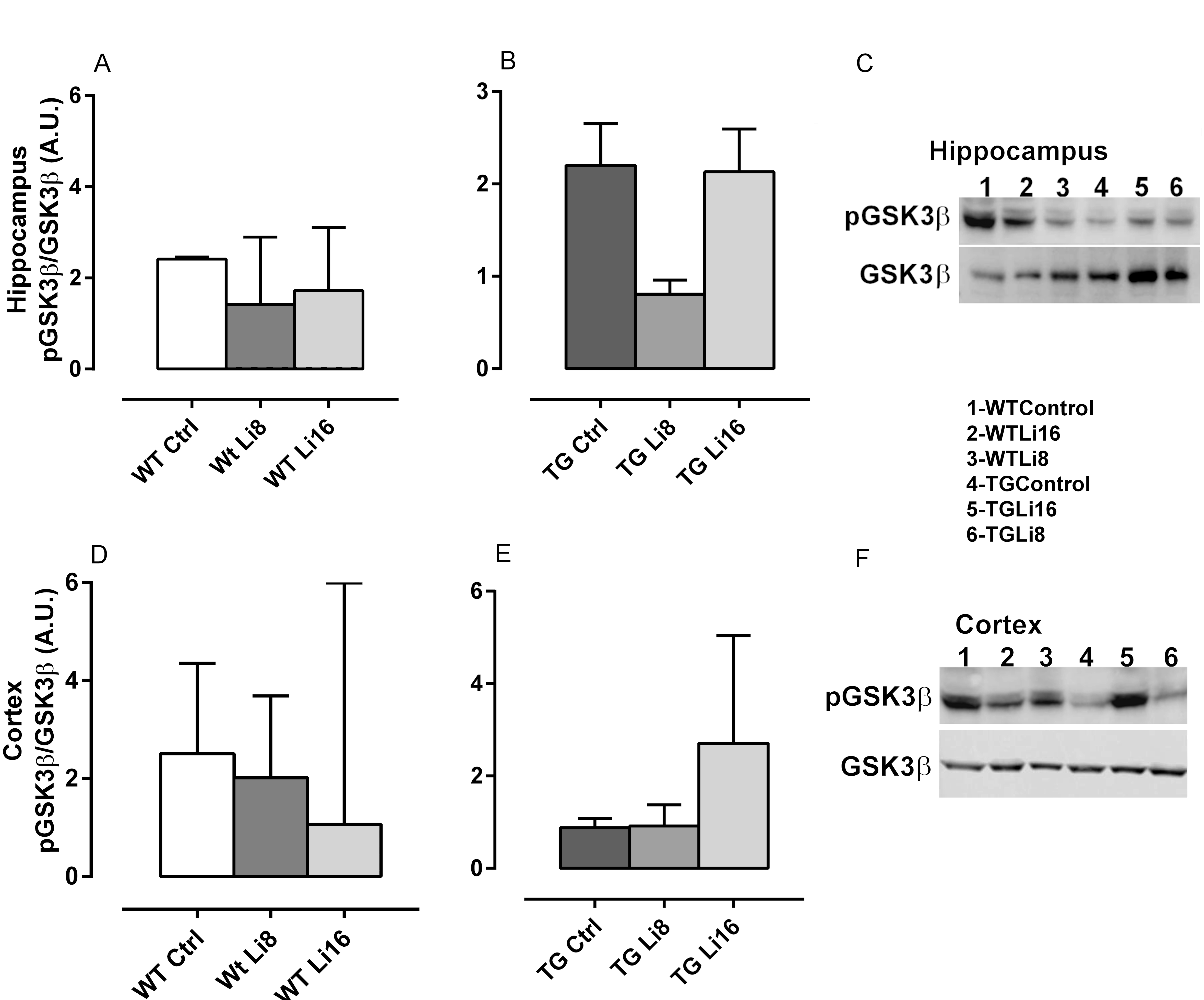

Supplement: S9 Fig — GSK-3β activity was measured by the proportion of density of phosphorylated (inactive) form and the total density of the enzyme. There was no difference in GSK-3β activity between WT Ctrl and TG Ctrl animals. In the same way, microdose lithium treatment did not change the GSK-3β activity in WT or TG mice. (TIF) [file pone.0142267.s009.tif]
